# Mechanistic insights into the noncovalent inhibition of SARS-CoV-2 PLpro: a multiscale computational study

**DOI:** 10.1007/s10822-026-00763-z

**Published:** 2026-02-05

**Authors:** Flávio Vinícius da Silva Ribeiro, Renan Patrick da Penha Valente, Hendrik G. Kruger, Jéssica de Oliveira Araújo, José Rogério A. Silva

**Affiliations:** 1 Graduate Program in Chemistry, Institute of Exact and Natural Sciences, Federal University of Pará, Belém, 66075-110 Brazil; 2 Laboratory of Computer Modeling of Molecular Biosystems (CompMBio), Federal University of Pará, Belém, PA 66075-110 Brazil; 3 Catalysis and Peptide Research Unit, University of KwaZulu-Natal, Durban, 4000 South Africa; 4 Institute of Chemistry, Universidade Estadual de Campinas (UNICAMP), Campinas, SP Brazil; 5 Center for Computational Engineering & Science, Universidade Estadual de Campinas (UNICAMP), Campinas, SP Brazil

**Keywords:** SARS-CoV-2 pLpro, Non-Covalent inhibition, BL2 loop, Conformational dynamics, Free energy profiling, Interaction hotspot mapping, Structure-Based drug discovery

## Abstract

The papain-like protease of SARS-CoV-2 (PLpro2) is integral to viral polyprotein cleavage and the modulation of host immune responses, positioning it as a critical target for antiviral drug development. Here, we elucidate the molecular mechanisms governing the noncovalent inhibition of PLpro2 through a comprehensive computational approach, including molecular docking, extensive molecular dynamics (MD) simulations, binding free energy calculations (MM/GBSA and SIE), principal component and free energy landscape (PCA/FEL) analyses, and protein–ligand interaction fingerprinting (ProLIF). We assessed a structurally diverse set of noncovalent inhibitors for their capacity to induce conformational rearrangements and stabilize key structural motifs of PLpro2, with particular emphasis on the BL2 loop. Notably, XR3 and A19 exhibited superior experimental and predicted binding affinities, which can be attributed to favorable contacts with essential residues Tyr268 and Gln269, the attenuation of loop dynamics, and the stabilization of energetically favorable conformational states. By contrast, less potent inhibitors were associated with increased conformational heterogeneity, fragmented free energy landscapes, and diminished interactions with critical loop residues. Therefore, our integrative analysis delineates the structural and energetic determinants underpinning noncovalent PLpro2 inhibition, underscoring the central roles of loop immobilization and π-stacking interactions in the rational design of next-generation PLpro2 inhibitors.

**Supplementary Information:**

The online version contains supplementary material available at 10.1007/s10822-026-00763-z.

## Introduction

The COVID-19 pandemic, triggered by the novel severe acute respiratory syndrome coronavirus 2 (SARS-CoV-2), has had a profound impact on global public health systems and socioeconomic stability [1, 2]. Originating in Wuhan, China, SARS-CoV-2 rapidly spread worldwide [3], with over 778 million confirmed cases reported by the World Health Organization as of June 29, 2025 (https://data.who.int/dashboards/covid19/cases, accessed July 20, 2025). The virus primarily spreads through respiratory droplets and close interpersonal contact, showing a strong affinity for the human respiratory tract [4]. The rapid viral replication during the pandemic led to the emergence of multiple variants and subvariants, many of which exhibited enhanced transmissibility and partial immune escape. Researchers worldwide have identified several variants of concern, including Alpha, Beta, Gamma, Delta, and Omicron [5–7].

At the molecular level, SARS-CoV-2 is a positive-sense, single-stranded RNA virus approximately 30 kb in length, sharing approximately 79% genome identity with SARS-CoV and approximately 50% with MERS-CoV [8]. Its genome encodes fourteen functional open reading frames (ORFs), producing structural, nonstructural, and accessory proteins. The 5′-terminal region comprises ORF1a and ORF1b, which encode sixteen nonstructural proteins (nsps) essential for viral replication and transcription. The 3′-terminal region encodes the structural proteins—spike (S), envelope (E), membrane (M), and nucleocapsid (N)—along with several accessory proteins [9, 10].

A critical step in SARS-CoV-2 infection is mediated by the spike (S) glycoprotein, which facilitates viral entry through high-affinity binding to the host angiotensin-converting enzyme 2 (ACE2) receptor [11, 12]. Post-entry, the viral polyproteins are processed by two essential cysteine proteases: the main protease (Mpro, also known as 3CLpro) and the papain-like protease (PLpro) [13]. Mpro, derived from nsp5, catalyzes proteolytic cleavage at eleven conserved sites between nsps 4–16, while PLpro, located within nsp3, cleaves the viral polyprotein at three specific junctions—nsp1/2, nsp2/3, and nsp3/4—thereby liberating nsps crucial for the replication-transcription complex [14, 15]. 

PLpro is situated between the SARS-unique domain (SUD) and the nucleic acid-binding domain (NUB) within nonstructural protein 3 (nsp3), one of the largest viral nonstructural proteins, with a molecular mass of approximately 212 kDa and comprising 1945 amino acid residues [16, 17]. PLpro exhibits a marked preference for cleaving peptide bonds following the conserved P4–P1 sequence motif Leu-X-Gly-Gly, where X represents Lys, Arg, or Asn [14, 18, 19]. This recognition motif is highly conserved across SARS-CoV-2, SARS-CoV, and MERS-CoV, underscoring its functional importance [14]. 

Beyond its critical role in viral polyprotein processing, SARS-CoV-2 PLpro (PLpro2) also functions as a deubiquitinase and deISGylase, removing post-translational modifiers such as ubiquitin (Ub) and interferon-stimulated gene 15 (ISG15) from host proteins [20, 21]. Ub is a conserved regulatory protein in eukaryotic systems [22], while ISG15 is a ubiquitin-like modifier composed of two Ub-like domains connected by a short peptide linker. Both proteins contain a conserved LXGG motif at their C-termini, which is specifically recognized and hydrolyzed by PLpro. Through removing Ub and ISG15, PLpro antagonizes host antiviral immune responses, particularly interferon-mediated signaling, thereby promoting viral evasion and replication. This dual enzymatic activity makes PLpro a strategic target for antiviral drug discovery efforts [23, 24]. Notably, proteases involved in viral polyprotein cleavage have been successfully targeted [25–27]. in the treatment of infections caused by HIV [28] and HCV, [29] providing a precedent for PLpro-targeted therapeutic development.

Despite its relevance, PLpro2 remains underexploited in the discovery of antiviral drugs [30, 31]. Currently, only two antiviral drugs have received full approval for the treatment of COVID-19: Veklury (remdesivir), which targets the viral RNA-dependent RNA polymerase (RdRp), and Paxlovid (a combination of nirmatrelvir and ritonavir), which inhibits the main protease (Mpro). [32–34]. Lagevrio™ (molnupiravir), another RdRp inhibitor, is available under emergency use authorization but has not been fully approved by the US FDA [35]. No antiviral agents targeting PLpro have been approved, underscoring the urgent and critical need to identify selective inhibitors with favorable pharmacokinetic and safety profiles.

Given the absence of clinically approved PLpro-targeting drugs, drug repurposing has emerged as an attractive strategy. This approach provides a rapid and cost-effective alternative to traditional drug discovery pipelines, which are often time-consuming and resource-intensive [36, 37]. Several compounds currently under investigation for PLpro inhibition are repurposed drugs. Among them, GRL0617 (5-amino-2-methyl-N[(1R)-1-naphthalen-1-ylethyl]benzamide) stands out as one of the most extensively studied noncovalent inhibitors [38, 39]. Initially developed against SARS-CoV PLpro, GRL0617 was found to retain its inhibitory activity against PLpro2, owing to the structural and functional homology between the two proteases [38, 40]. Previous studies, such as Ferreira et al. [41], have demonstrated that noncovalent inhibitors can induce significant conformational changes in PLpro2, particularly within the BL2 loop region, which acts as a regulatory gate for substrate access and inhibitor binding..

In this context, computational chemistry techniques such as molecular docking [42–44] and molecular dynamics (MD) simulations [45–47] have become essential tools in the design and optimization of antiviral agents. These methods provide valuable insights into protein–ligand interactions, conformational changes, and binding free energies. Several small molecules that inhibit PLpro2 have been identified through drug design and repurposing efforts. One common reference inhibitor is GRL0617, a noncovalent molecule with an IC₅₀ of 2.20 μM. [19] More active molecules have been developed, such as Jun9-72–2 (JU2, IC₅₀ = 0.67 μM), Jun9-75–4 (JU4, IC₅₀ = 0.62 μM) [48] and Analogue 19 (A19, IC₅₀ = 0.44 μM) [49]. Other strong candidates include Rac5c (RC5) and XR8-series inhibitors like XR8-23 (XR3, IC₅₀ = 0.39 μM) and XR8-24 (XR4, IC₅₀ = 0.56 μM) [50]. In comparison, repurposed drugs such as YM155 (YM1, IC₅₀ = 2.47 μM) [51], cryptotanshinone (CRY, IC₅₀ = 5.63 μM) and disulfiram (DIS, IC₅₀ = 7.52 μM) [52] are less effective. These various inhibitors, from new designs to existing drugs, help us study how molecules block the action of PLpro2. Therefore, in this study, we employed molecular docking, MD simulations and free energy calculations to explore the structural basis of the noncovalent inhibition of PLpro, aiming to support drug repurposing efforts and guide the rational development of new therapeutic candidates.

## Material and methods

### System setup for molecular docking

The crystal structure of the PLpro enzyme in complex with the inhibitor XR8-24 was retrieved from the Protein Data Bank (PDB ID: 7LBS), which was resolved at a resolution of 2.80 Å. [50] Initial preparation of the protein structure involved removing crystallographic water molecules and non-amino acid residues, followed by the addition of hydrogen atoms using the UCSF Chimera program (version 1.17.3) [53]. The catalytic dyad, Cys111–His272, was modeled in its neutral form, while Asp286 was assigned a deprotonated state, consistent with its expected ionization at physiological pH. To validate the docking protocol, redocking of the native ligand (XR8-24) was performed using six different molecular docking programs: GOLD (Genetic Optimization for Ligand Docking) (v. 2022.3.0) [54], HYBRID (v. 4.2.01) [55], AutoDock (v. 4.2.6) [56], AutoDock Vina (v. 1.2.3) [57], DOCK (v. 6.10) [58] and Molegro Virtual Docker (MVD) (v. 5.5) [59]. The redocking accuracy of each software was assessed by calculating the RMSD between the predicted and crystallographic poses of XR8-24. In GOLD, the GoldScore [54] and ChemPLP [60] scoring functions were applied, using a 10 Å radius around the ligand binding site. HYBRID employed the Chemgauss4 scoring function [55, 61], while AutoDock used the AD4 scoring protocol [62]. AutoDock Vina applied its default Vina scoring function [57]. DOCK utilized the Grid score [63] and MVD relied on the MolDock scoring algorithm [64]. In all cases, the docking search space was centered on the crystallographic coordinates of XR8-24 (X = 9.942 Å, Y = −12.256 Å, Z = 32.740 Å). Following RMSD-based validation, a second criterion was used to calculate the enrichment factor (EF) for each crystallographic complex [65–67]. This metric was used to evaluate the ability of each docking protocol to prioritize active compounds over decoys. The objective was to identify a convergence between high EF values and superior experimental activity, as indicated by lower average half-maximal inhibitory concentration (IC₅₀) values. This approach supports the selection of docking protocols with greater predictive reliability in a consensual docking framework [68, 69]. EF value can be calculated as (Eq. 1): [70].

1 $${EF}_{\%}=\frac{{Hits}_{sel}}{{Hits}_{tot}}\times \frac{{NC}_{tot}}{NC}$$

where Hits_sel_ represents the number of known active compounds retrieved within the top 20% of the ranked list, Hits_tot_ is the total number of known actives in the dataset, NC is the number of compounds in the top 20% subset and NC_tot_ denotes the total number of compounds screened. Here, the EF was calculated at the 20% threshold (EF_20%_) to evaluate the effectiveness of docking and consensus scoring in prioritizing most potent PLpro2 inhibitors.

Post-docking analyses were conducted using LigPlot +  [71] to visualize and characterize hydrogen bonding and hydrophobic interactions between the docked ligands and key amino acid residues in the PLpro2 active site. The same docking protocol was applied to non-crystallographic inhibitors and additional crystallographic complexes (PDBs 7CMD [19], 7SDR [48] and 7D7L [51]), ensuring methodological consistency across all systems evaluated (Table 1).

**Table 1:** Summary of PLpro2 inhibitors evaluated in this study, including names, corresponding PDB codes (when available), 2D chemical structures, assigned abbreviations, and experimentally determined IC₅₀ values (μM)

Name	PDB code	2D structure	Abbreviation	IC_50_ (μM)
GRL0617	7CMD		GRL	2.20 [19]
Jun9-72–2	7SDR		JU2	0.67 [48]
Jun9-75–4	–		JU4	0.62 [48]
Analogue 19	–		A19	0.44 [49]
Rac5c	–		RC5	0.81 [40]
XR8-23	–		XR3	0.39 [50]
XR8-24	7LBS		XR4	0.56 [50]
YM155	7D7L		YM1	2.47 [51]
Cryptotanshinone	–		CRY	5.63 [51]
Disulfiram	–		DIS	7.52 [52]

### Molecular dynamics (MD) simulation

Classical MD simulations were employed to explore the atomic-level dynamics of PLpro2 and its complexes with selected inhibitors (Table 1), using the AMBER22 software [72]. Simulations were performed using the PMEMD module [73] for optimal computational efficiency. Electrostatic potential-derived atomic charges for the inhibitors were computed at the Hartree–Fock (HF) level using the 6-31G** basis set [74], followed by the Restrained Electrostatic Potential (RESP) fitting procedure [75], as implemented in the Gaussian09 software [76]. The protonation states of enzyme residues at physiological pH (7.0) were determined using PROPKA 3.1 [77]. Classical parameterization was conducted using the General AMBER Force Field (GAFF) [78] for the inhibitors and the ff19SB force field [79] for the protein. Zn(II) coordination in the PLpro2 zinc-finger (ZnF) motif was treated using the ZAFF parameter set [80]. System preparation, including topology and coordinate file generation, was carried out using the tLeap module of AMBER. Each PLpro2 system was solvated in a cubic box of TIP3P water molecules [81] extending 12 Å beyond the solute in all directions. Chloride counterions (Cl^−^) were added to neutralize the overall charge of the system. Energy minimization, heating, and equilibration protocols were executed using the SANDER module from AmberTools [82]. The minimization procedure consisted of four sequential stages, totaling 25,000 steps. The first stage employed 10,000 steps of the steepest descent algorithm, followed by 15,000 steps of the conjugate gradient method. Each subsequent stage involved 10,000 steps using the same approach. The systems were then gradually heated from 0 to 310 K under constant volume conditions (NVT ensemble), using the Langevin thermostat [83] and a positional restraint force constant of 25 kcal/(mol·Å^2^) applied to heavy atoms. Long-range electrostatic interactions were treated using the Particle Mesh Ewald (PME) method [84] with a 9 Å cutoff for nonbonded interactions. Bond lengths involving hydrogen atoms were constrained using the SHAKE algorithm [85] and the equation of motion was integrated every 2 fs using the Verlet algorithm [86]. 

The production MD simulations were conducted under isothermal–isobaric conditions (NPT ensemble) at 310 K and 1 atm, using the PMEMD module. Each system was simulated in triplicate for 500 ns, resulting in a total simulation time of 1.5 μs per system.

### Structural and free energy landscape (FEL) analysis

The structural analysis of PLpro2 dynamics was performed using the CPPTRAJ module [87] of the AmberTools [82]. Cα atom trajectories were extracted from the 1.5 µs of MD simulations and subjected to principal component analysis (PCA) to characterize the dominant collective motions of the system. PCA identifies orthogonal eigenvectors (principal components, PCs) that describe the most significant variance in atomic displacements over time. The first principal component (PC1) typically captures the most significant conformational fluctuation and often corresponds [88, 89] to the biologically relevant motions of the protein.

An iterative RMSD-based alignment procedure was employed to superimpose the MD snapshots, ensuring the robustness of the PCA. This process excluded highly flexible or structurally deviant regions to avoid distorting the alignment. This approach refines the identification of structurally invariant "core" residues, which serves as a stable reference for PCA and reduces the underestimation of accurate atomic displacements [88–90]. 

To further characterize the conformational space sampled by PLpro2, a free energy landscape (FEL) analysis was performed. The FEL provides a statistical mechanics-based depiction of the protein’s potential energy surface, identifying energetically favorable conformational states and the transitions between them [91, 92]. Unlike traditional structural analysis, which emphasizes discrete conformational snapshots, the FEL captures a continuum of states, thereby offering a more comprehensive view of the protein's functional conformational ensemble [93]. 

In this study, the FEL was constructed using the first two principal components (PC1 and PC2), according to the following expression (Eq. 2):

2 $$\Delta G\left({PC}_{s}\right) =-{k}_{B}Tln\left(\frac{P({PC}_{1},{PC}_{2})}{{P}_{max}}\right)$$

where $\Delta G\left({PC}_{s}\right)$ is the relative free energy, P(PC_1_,PC_2_) is the joint probability distribution of the conformations projected onto PC_1_ and PC_2_, P_max_ is the maximum probability observed in the distribution, *k*_*B*_​ is the Boltzmann constant, and T is the absolute temperature (set to 310 K) [94]. This formulation normalizes the landscape such that the most populated state has a free energy of zero, facilitating direct interpretation of energy minima.

The resulting free energy surface reveals distinct basins corresponding to the most probable conformational states, Min1 and Min2. These minima likely represent structurally stable, functionally relevant conformations of PLpro2. Although explicit transition states between Min1 and Min2 may not be readily resolved from this analysis, intermediate (IN) conformations can be inferred from adjacent local minima, providing insight into potential conformational pathways along the FEL.

### Binding free energy calculations

Binding free energy (ΔG_bind_) calculations were performed using two end-point methods: the Molecular Mechanics/Generalized Born Surface Area (MM/GBSA) [95, 96] and the Solvated Interaction Energy (SIE) methods [97–99]. For both methods, the final 100 ns of each MD simulation replica (a total of 300 ns) were used to ensure energetic convergence and system stability.

MM/GBSA calculations were done using the AmberTools suite's *MMPBSA.py* module [100]. The binding free energy was estimated based on the following thermodynamic cycle (Eq. 3):

3 $$\Delta {G}_{bind}={G}_{complex}-{G}_{receptor}- {G}_{ligand}$$

where G_complex_, G_receptor_ and G_ligand_ represent the free energies of the complex, receptor, and ligand, respectively. The enthalpic contribution to ΔG_bind_ was decomposed into molecular mechanics and solvation terms as (Eq. 4):

4 $$\Delta {G}_{bind}=\Delta H-T\Delta S \approx \Delta {E}_{MM}+ \Delta {G}_{sol}-T\Delta S$$

5 $${\Delta E}_{MM}=\Delta {E}_{int}+ \Delta {E}_{elec}+ \Delta {E}_{vdW}$$

6 $$\Delta {G}_{sol}=\Delta {G}_{GB}+ \Delta {G}_{nonpolar}$$

7 $$\Delta {G}_{nonpolar}=\gamma \cdot SASA$$

where $\Delta H$ represents the enthalpy contribution of the system, $T\Delta S$ is the entropic term, which was excluded to reduce computational complexity; $\Delta {E}_{MM}$ consists of the internal energy ($\Delta {E}_{int}$), electrostatic ($\Delta {E}_{elec}$) and van der Waals interactions ($\Delta {E}_{vdW}$). The solvation free energy ($\Delta {G}_{sol}$) is the sum of the polar contribution from the Generalized Born (GB) method ($\Delta {G}_{GB}$) and the nonpolar contribution ($\Delta {G}_{nonpolar}$), calculated by multiplying the surface tension (γ) by the solvent-accessible surface area (SASA).

The SIE method [99] was applied using the SIETRAJ package [97] and ΔG_bind_ was computed as (Eq. 8):

8 $${\Delta G}_{SIE}=\alpha \left({E}_{coul}+{E}_{vdW}+{E}_{RF}+{E}_{nonpolar}\right)+C$$

In this expression, E_coul_ and E_vdW_ represent Coulombic and van der Waals interactions within the bound complex, E_RF_ accounts for polar desolvation, and E_nonpolar_ reflects nonpolar solvation. The empirical parameters α = 0.1048 and C = –2.89 kcal/mol were adopted from prior optimization on a standard dataset of 99 protein–ligand complexes [97]. 

Both methods are widely employed in drug discovery to estimate the binding affinities of small molecules to biomolecular targets. They offer a balance between speed and accuracy compared to more complex free energy calculation techniques such as Free Energy Perturbation (FEP) and Thermodynamic Integration (TI) [96]. Implementing these methods facilitate the evaluation of binding free energy values associated with the PLpro2 enzyme through MD simulations.

### Interaction fingerprint (IFP) analysis

Protein–ligand interaction fingerprints (IFPs) are compact binary descriptors that capture the presence or absence of specific noncovalent interactions between a ligand and the residues in a protein's binding site, offering a simplified one-dimensional representation of complex 3D molecular interactions [101]. Here, IFPs were generated to characterize the dynamic interactions between inhibitors and the binding site residues of PLpro2 using representative conformations of MD simulations. The ProLIF (Protein–Ligand Interaction Fingerprints) library systematically computed these fingerprints [102]. The interaction matrix accounted for key interaction types, including hydrophobic, polar, aromatic, hydrogen bond acceptors, and hydrogen bond donors. An interaction was recorded when any heavy ligand atom was within 4.0 Å of a residue in the PLpro2 binding site.

## Results and discussion

### Structural basis of catalysis and inhibition in PLpro2: molecular docking and consensus analysis

**Fig. 1:**
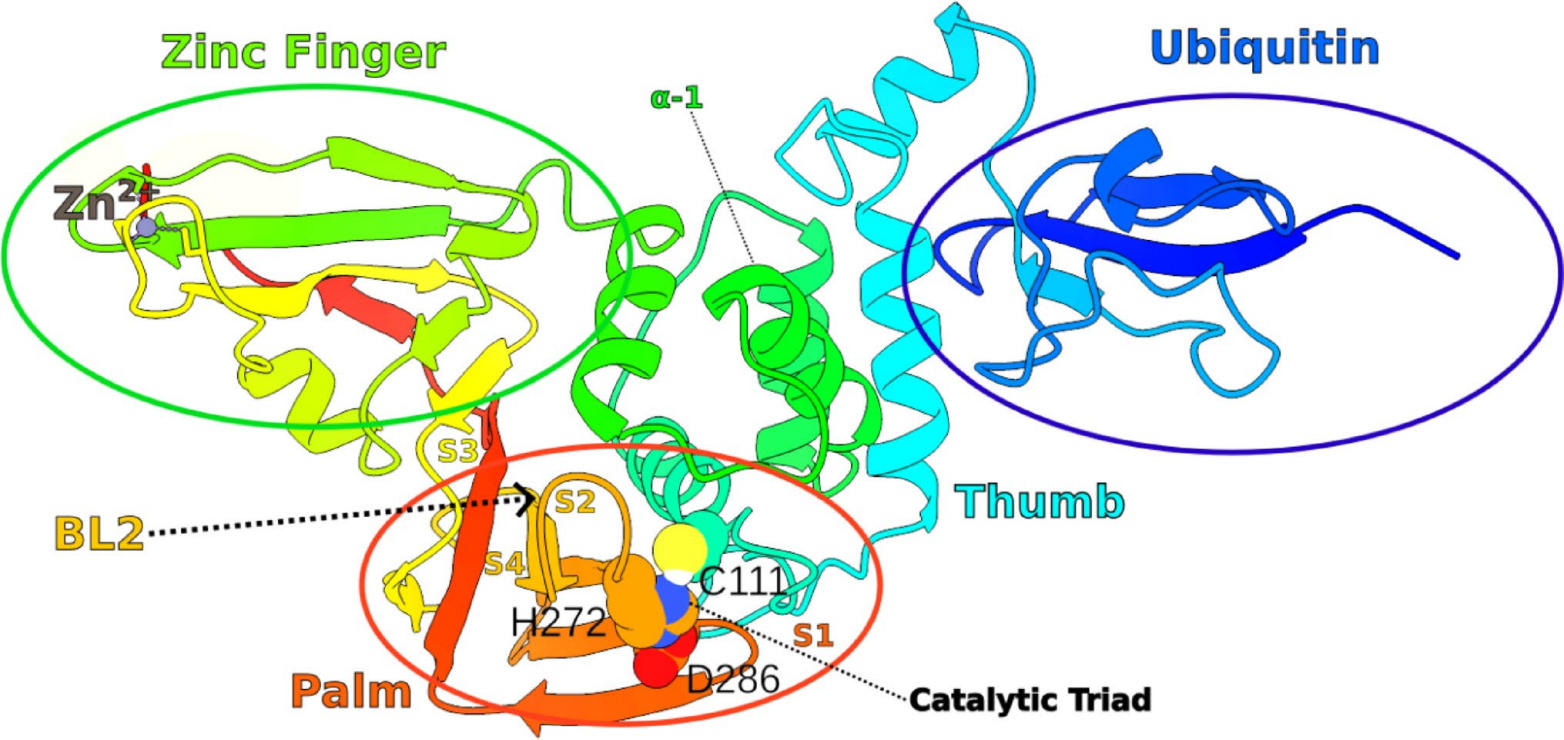
3D structure of PLpro2 and illustrates its key structural domains. The ubiquitin-like (Ub) domain is shown in blue, the palm domain in orange, and the ZnF domain in green. The catalytic triad residues (Cys111, His272, and Asp286) are represented as spheres and labeled. A detailed description of its secondary structure is included as Fig. S6

As highlighted previously, PLpro is a protease expressed as part of the Nsp3 multidomain protein and contains a catalytic triad composed of the residues Cys111, His272, and Asp286. The monomeric structure of PLpro is organized into three distinct domains: ubiquitin-like (Ub), palm, and ZnF (Fig. 1). The catalytic triad is situated within the palm domain, immediately below the sub-region known as the blocking loop (BL2). A Zn(II) ion, essential for the structural stability of the protein, is coordinated in a tetrahedral geometry by four cysteine residues within the zinc finger domain [103, 104]. Noncovalent inhibition assays have identified a binding site near the BL2, with key interactions involving residues Gly266, Asp267, Tyr268, Gln269, and Cys270. Ligand binding at this site is believed to sterically hinder access to the catalytic triad, effectively suppressing enzymatic activity [24].

**Fig. 2:**
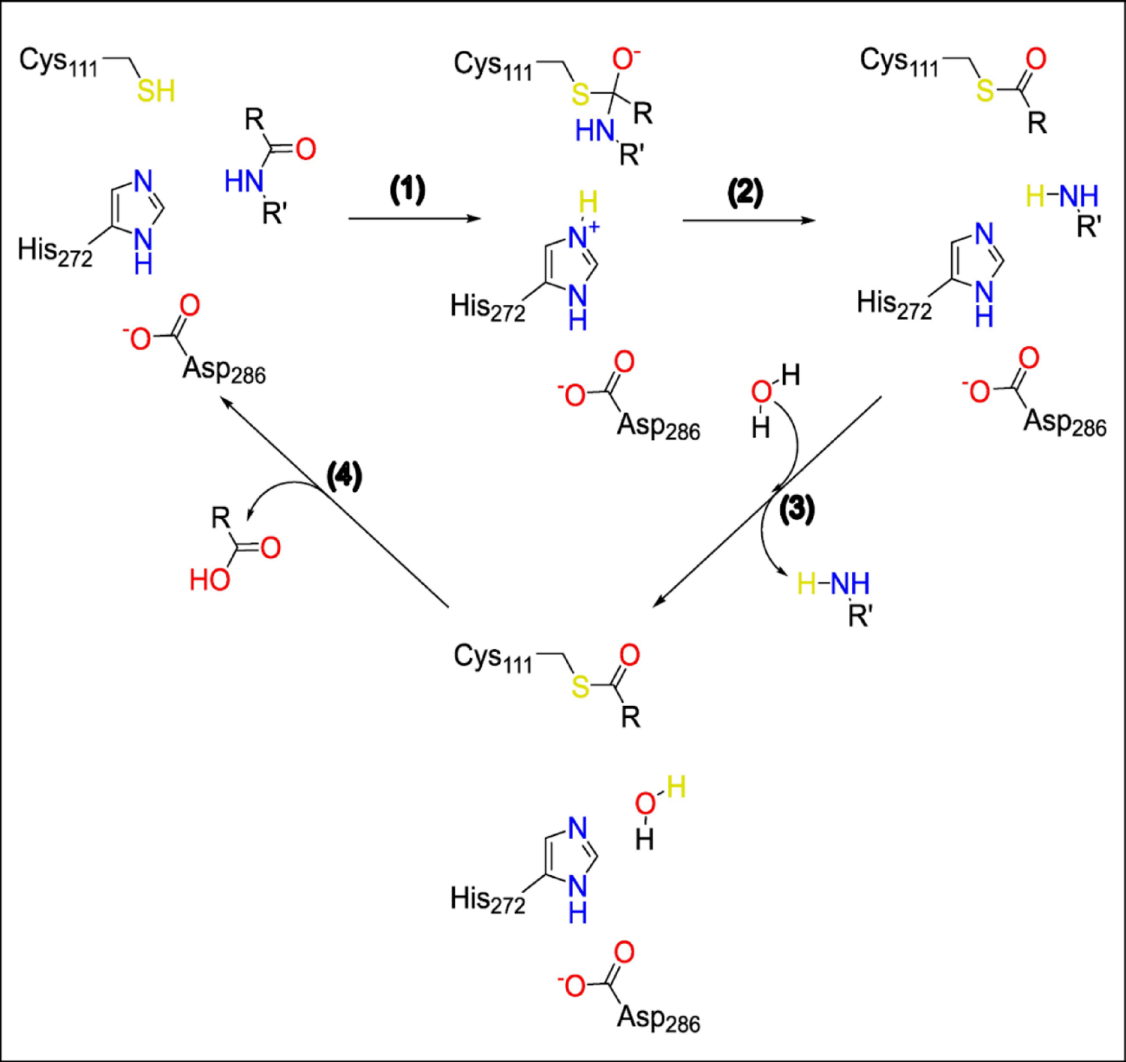
Schematic representation of the catalytic mechanism of PLpro2, highlighting the acylation and deacylation steps. The figure illustrates (1) nucleophilic attack by Cys111 on the peptide bond of the substrate, (2) formation of the covalent acyl-enzyme intermediate, and (3) subsequent hydrolysis that releases the cleavage product and (4) regenerates the active site. This cycle underscores the role of the catalytic triad (Cys111, His272, Asp286) in peptide bond hydrolysis

A detailed understanding of the catalytic mechanism of PLpro2 is essential for the rational design of effective inhibitors. The catalytic cycle initiates with the deprotonation of the thiol group of Cys111, facilitated by the catalytic dyad His273 and Asp287. Upon substrate binding, a nucleophilic attack promoted by thiol group of Cys111 into carbonyl group of the substrate leads to the formation of a first tetrahedral intermediate, which undergoes cleavage upon entering a water molecule. This results in a second tetrahedral intermediate, which is subsequently hydrolyzed to release the final cleavage product, thereby regenerating the protonated state of Cys111 and completing the catalytic cycle (Fig. 2) [105].

The PLpro2 shares 83% sequence identity and 90% similarity with SARS-CoV PLpro (PLpro1), and 31% identity and 49% similarity with that of MERS-CoV, reflecting high evolutionary conservation within the SARS lineage and greater divergence from MERS-related coronaviruses [23]. Despite this high homology, subtle structural differences appear critical for maintaining the conformational stability and enzymatic activity of PLpro2. Key amino acid substitutions distinguishing PLpro2 from PLpro1 include Thr75(Leu), Pro129(Ala), Thr172(His), Lys200(Thr), Lys274(Thr), and Cys284(Arg), which may contribute to differences in protein flexibility, substrate recognition, and inhibitor susceptibility [23]. 

S3/S4 sites are key targets for drug discovery [24]. The primary inhibitory mechanism of ligands binding to these sites is preventing substrate entry into the active site. The binding pocket is predominantly hydrophobic, with residues Lys157, Leu162, Gly163, Asp164, Glu167, Met208, Pro248, Tyr264, Gly266, Asn267, Tyr268, Gln269, Tyr273, and Thr301 interacting with ligands within 3.5 Å. The abundance of non-polar and aromatic residues suggests that π-π or CH/NH-π interactions are the main forces driving ligand binding.

**Fig. 3:**
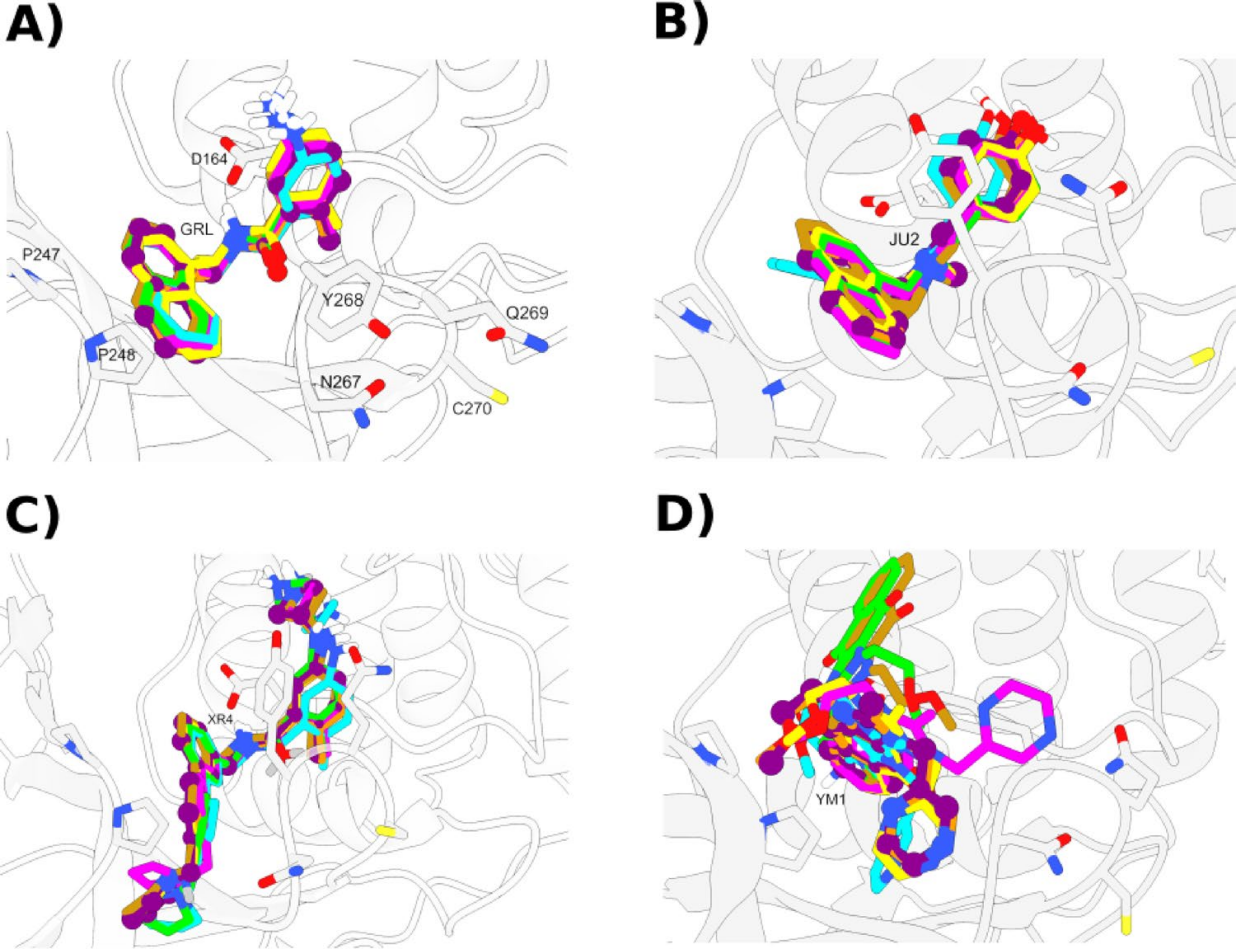
Consensus redocking of crystallographic inhibitors: **a** GRL, **b** JU2, **c** XR4, **d** YM1. Crystal poses are shown in purple (ball-and-stick), while predicted docking poses are color-coded by software: Vina (lime), AutoDock (cyan), MVD (dark orange), HYBRID (yellow), DOCK (dark goldenrod), and GOLD (magenta). RMSD values (in Å) are available as Supporting Information (SI), Table S1

In this study, molecular docking was utilized to predict the binding poses and affinities of selected noncovalent inhibitors targeting the active site of PLpro2. A panel of six docking programs (AutoDock, Vina, MVD, HYBRID, DOCK, and GOLD) was employed, and their normalized scores were systematically analyzed through correlation and enrichment metrics (Table 2). To validate docking accuracy, the lowest RMSD poses of crystallographically resolved inhibitors (GRL, JU2, XR4, and YM1) were compared and are illustrated in Fig. 3.

**Table 2:** Docking scores from six different programs and corresponding experimental pIC₅₀ (− logIC₅₀) values for ten PLpro2 inhibitors

System	Vina	AutoDock	MVD	HYBRID	DOCK	GOLD	pIC50	Fr*
XR3	− 7.69	− 10.94	− 154.75	− 15.99	− 60.98	75.81	6.41	0.40
A19	− 7.48	− 11.49	− 150.62	− 0.50	− 54.62	93.79	6.36	0.36
XR4	− 10.00	− 11.88	− 179.07	− 16.96	− 61.34	104.48	6.25	0.00
JU4	− 6.68	− 8.97	− 131.21	− 4.47	− 42.89	73.93	6.21	0.94
JU2	− 8.50	− 8.14	− 113.93	− 14.33	− 50.34	78.58	6.17	1.24
RC5	− 7.85	− 8.88	− 105.68	− 4.33	− 47.42	80.83	6.09	1.22
GRL	− 10.20	− 9.64	− 132.39	− 15.21	− 55.96	92.09	5.66	0.82
YM1	− 5.40	− 5.19	− 83.59	− 1.62	− 48.16	54.85	5.61	2.00
CRY	− 7.15	− 8.37	− 109.14	− 8.77	− 35.51	60.84	5.25	1.26
DIS	− 3.71	− 5.58	− 98.52	− 5.60	− 34.77	64.01	5.12	1.79

^*^Final ranking (Fr) is the sum of normalized docking scores from the best-performing scoring functions, AutoDock and MVD. Lower Fr values indicate more potent predicted inhibitors. The scores were obtained using Vina (kcal/mol), AutoDock (kcal/mol), MVD (kcal/mol), HYBRID (kcal/mol), DOCK (kcal/mol) and GOLD

According to the scoring values presented in Table 2, the best combination of docking scores, without weighting, was achieved using AutoDock and MVD. This combination yielded an R^2^ value of −0.71, indicating a suitable linear correlation with the experimental activity values (pIC₅₀). The resulting consensus ranking (Fr) is displayed in Table 2. Notably, the three most potent inhibitors (XR3, A19 and XR4) along with the three weakest compounds (YM1, CRY and DIS) are ranked appropriately based on their experimental performance. In addition to showing strong agreement with experimental pIC₅₀ values, the consensus approach achieved an EF_20%_ of 3.33, effectively prioritizing the most active compounds. These results collectively highlight the predictive power and practical utility of the consensus strategy in identifying potent PLpro2 inhibitors.

To further clarify the binding mode of noncovalent inhibitors to PLpro2, we analyzed protein–ligand interactions with a focus on key residues near the BL2 and the S3/S4 subpockets. Table 3 summarizes the hydrogen bonding and hydrophobic interactions for each complex. As anticipated, several inhibitors form hydrogen bonds with catalytically relevant residues such as Asp164, Tyr268, and Gln269, which are located adjacent to the catalytic triad and play a crucial role in stabilizing the ligands. Notably, XR3 and XR4, two of the most potent inhibitors, form hydrogen bonds with both Asp164 and either Gln269 or Gly266, which supports their strong experimental affinity (pIC₅₀ > 6.2). Similarly, A19 interacts with Asp164 and Tyr268 through hydrogen bonding, aligning with its robust inhibitory profile.

**Table 3:** Summary of protein–ligand interactions for PLpro2 inhibitor complexes

System	Hydrogen bonding interactions	Hydrophobic interactions
GRL	Asp164, Tyr268, Gln269	Lys162, Gly163, Pro247, Pro248, Tyr264, Tyr273, Thr301
JU2	Leu162	Gly163, Asp164, Glu167, Pro248, Tyr264, Asn267, Tyr268,Tyr273
JU4	Leu162	Gly163, Asp164, Pro248, Tyr264, Gly266, Asn267, Tyr268, Gln269, Tyr273, Thr301
RC5	–	Leu162, Gly163, Aps164, Pro247, Pro248, Tyr264, Tyr268, Gln269, Tyr273
A19	Asp164, Tyr268	Gly163, Asp164, Arg166, Glu167, Met208, Pro247, Pro248,Tyr264, Gly266, Asn267, Tyr268, Gln269, Tyr273
XR3	Asp164, Gly266	Leu162, Gly163, Glu167, Pro248, Tyr264, Tyr268, Gln269, Tyr273, Pro299, Thr301
XR4	Asp164, Gln269	Leu162, Gly163, Glu167, Pro248, Gly266, Tyr264, Asn267, Tyr268, Tyr273, Thr301
CRY	Gln269	Leu162, Gly163, Glu164, Pro248, Tyr264, Tyr268
YM1	–	Asp164, Pro247, Pro248, Tyr264, Gly266, Asn267, Tyr268, Tyr273, Thr301
DIS	–	Leu162, Gly163, Asp164, Pro247, Tyr264, Gly266, Asn267, Tyr268, Gln269

Hydrogen bond donors or acceptors (left column) and hydrophobic contacts (right column) are listed for each ligand, based on key interactions with residues within the binding pocket. Only residues within 3.5 Å of the ligand were considered. A 2D interaction diagram is available as SI (Figure S1)

Hydrophobic interactions, particularly with residues Leu162, Gly163, Pro248, Tyr264, Tyr268, and Tyr273, are prevalent among the most active compounds (XR3, XR4, A19, GRL and JU4). This highlights the importance of nonpolar interactions in stabilizing ligands within the binding cleft. The presence of multiple aromatic residues in the binding site likely promotes π–π or CH/π interactions, as previously suggested. In contrast, weaker inhibitors such as CRY, YM1, and DIS display fewer hydrogen bonds, relying primarily on hydrophobic interactions, many of which are restricted to peripheral residues such as Pro247, Pro248 and Tyr264. These interaction profiles further validate their lower experimental activity (pIC₅₀ < 5.7) and reaffirm the importance of deep-pocket engagement and multi-residue hydrogen bonding for effective inhibition of PLpro2.

Our docking analysis shows that most inhibitors, such as GRL, JU2, and XR4, bind to the BL2 allosteric pocket, while YM1 binds to a different allosteric site near the ZnF. We used crystal structures to accurately place known ligands. For similar inhibitors without crystal structures, we relied on consistent structure–activity relationships and docking results in the BL2 site. These findings give a clear structural basis for our later MD and free energy analysis.

### MD, PCA and FEL analysis

Molecular docking is a fast and efficient computational approach for predicting ligand binding poses and relative affinities, but it typically considers the protein as a rigid entity and simplifies solvation and entropy effects [106–108]. Consequently, docking scores may not represent actual binding affinities or capture conformational changes, such as the flexibility of the BL2 loop in PLpro2, which is important for ligand recognition. To address these limitations, MD simulations were conducted to examine the conformational landscape and stability of the protein–ligand complexes, providing more accurate information on binding energetics and mechanisms.

Then, based on the docking results and experimental IC_50_ values, four representative PLpro inhibitors were selected for MD analysis: the two most potent inhibitors (XR3 and A19) and the two least potent (CRY and DIS) (see Table 1). The remaining compounds were included in the SI file (Figures S2, S3 and S4). Notably, YM1 exhibits a dual binding mode, as indicated by experimental structural data [51] and was therefore included for detailed analysis and discussion.

**Fig. 4:**
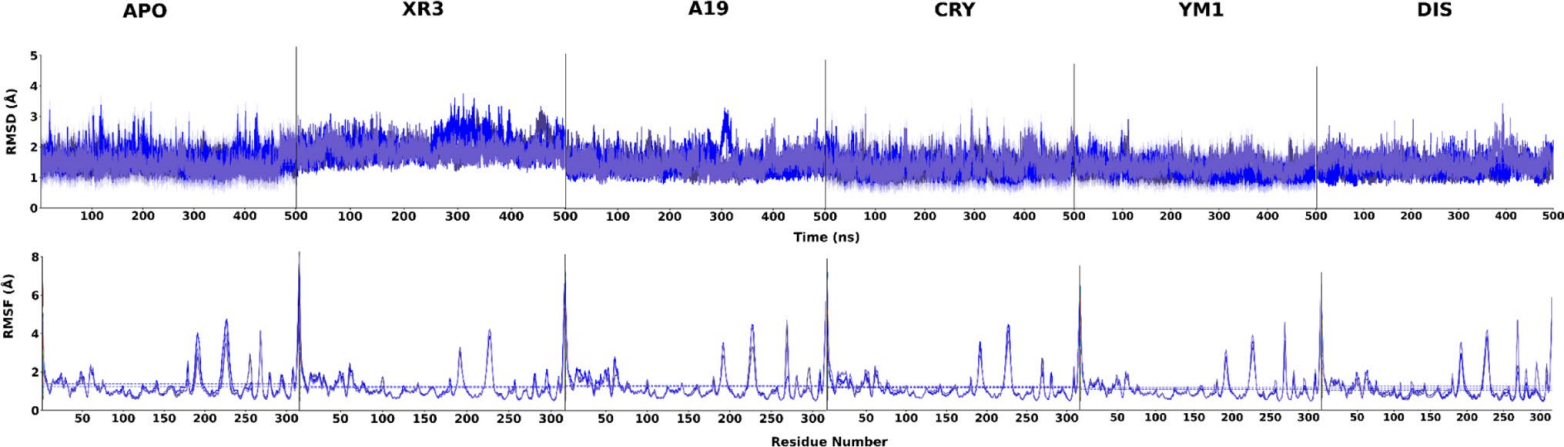
RMSD and RMSF plots of for APO, XR3, A19, CRY, YM1 and DIS systems (other PLpro2 inhibitors are available as SI, Figure S2 and S3). All plots reflect triplicate results

Initially, to comprehensively assess the structural stability and dynamic behavior of PLpro2 upon ligand association, RMSD and RMSF analyses were conducted on the protein backbone for all simulated complexes (Fig. 4 and Table S2). The apo form displayed the highest RMSD (1.88 ± 0.33 Å), indicative of increased structural heterogeneity in the absence of ligand engagement. Conversely, ligand-bound complexes exhibited reduced and stabilized RMSD values (1.37–1.57 Å), supporting the hypothesis that ligand binding constrains the conformational dynamics of PLpro2.

RMSF analysis corroborated these findings. The apoform demonstrated elevated average residue fluctuations (1.29 ± 0.79 Å), predominantly within the loop and peripheral regions. Ligand-bound complexes exhibited attenuated per-residue fluctuations overall, with JU2 (1.17 ± 0.74 Å) and XR3 (1.18 ± 0.71 Å) achieving the lowest average RMSF values. Notably, XR4 exhibited the highest RMSF (1.38 ± 0.83 Å) among the complexes, indicating enhanced residue-level mobility despite a moderately stabilized global RMSD.

In combination, these results demonstrate that ligand association diminishes both global and local flexibility in PLpro2, with inhibitors such as JU2, JU4, and A19 imparting marked structural stabilization. The discordance observed for XR3—elevated RMSD concurrent with low RMSF—may reflect localized stabilization within critical regions (e.g., the BL2 loop), concomitant with sustained flexibility in other domains. These structural dynamics are consistent with subsequent PCA and FEL results, highlighting the significance of conformational restriction in the efficacy of noncovalent PLpro2 inhibition.

**Fig. 5:**
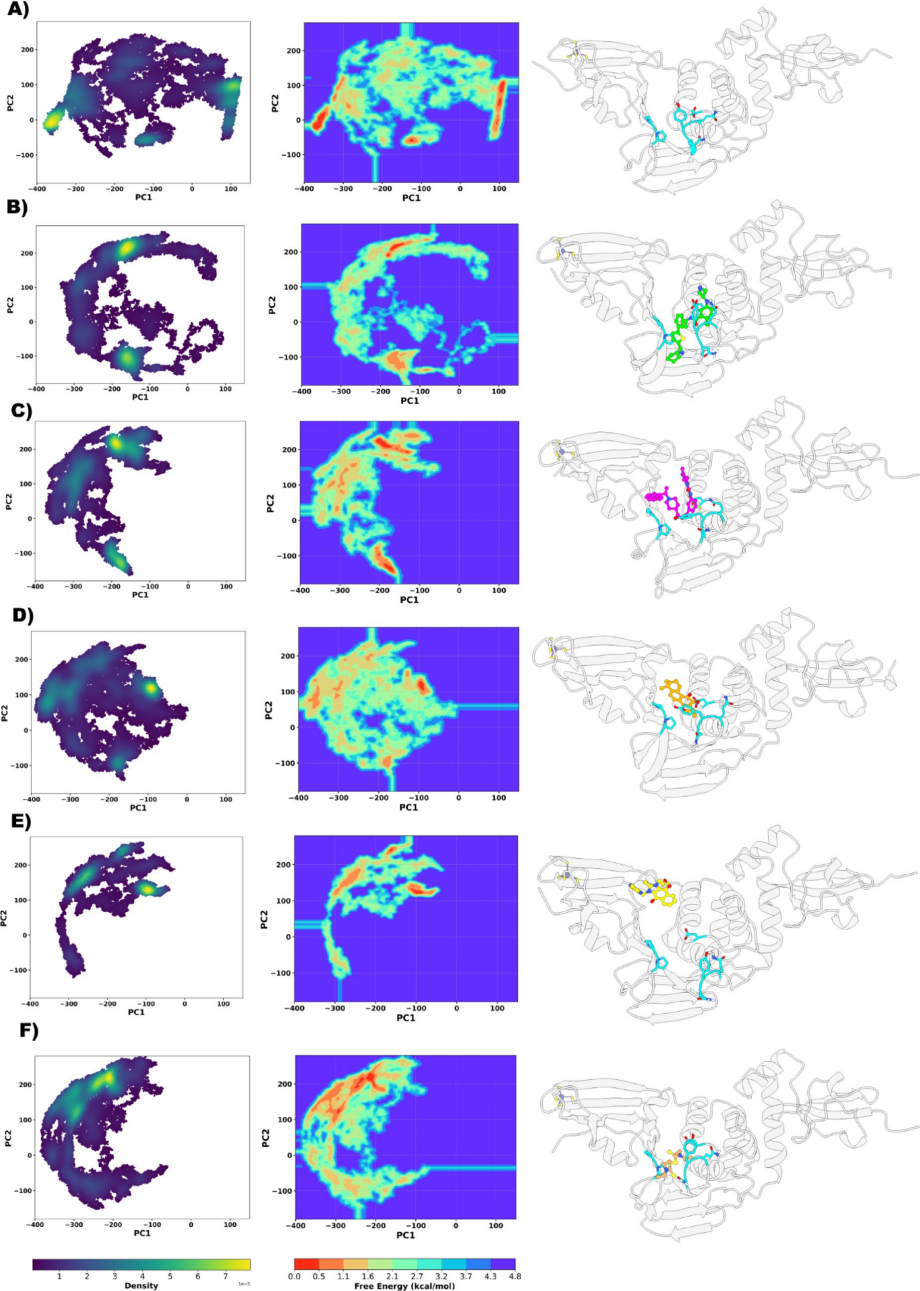
Principal component analysis (PCA, left) and free energy landscape (FEL, center) plots for the PLpro2 systems: **A** APO, **B** XR3, **C** A19, **D** CRY, **E** YM1, and **F** DIS. The representative structure corresponding to the global minimum of each FEL is shown on the right

To improve the understanding of the collective and individual dynamic behaviors of the PLpro2 systems, PCA and FEL analyses were performed across all MD trajectories (Fig. 5). Protein motions were extracted from the combined 1.5 µs of simulation data (3 replicas × 500 ns each), including both the apo form and the complexes with noncovalent inhibitors. To ensure a consistent comparison among systems, a unified PCA was carried out using the aggregated trajectories, allowing the identification of common PCs that capture the dominant collective motions of PLpro. The first two PCs explained approximately 61% of the total variance in the combined analysis of all systems, and were therefore used to project the trajectories, enabling a consistent comparative visualization of the conformational space sampled by each complex.

Overall, in the absence of ligand, the apo system exhibits PC1 dominance (> 72%), indicative of highly directional and unconstrained BL2 loop motions. Potent BL2-site inhibitors, such as XR3 (PC1: 20.00% and PC2: 57.70%) and A19 (PC1: 13.08% and PC2: 42.77%), redistribute the dominant variance toward PC2, signifying ligand-induced stabilization and restriction of alternative conformational states. Weaker inhibitors (CRY and DIS) result in a more equal variance distribution between PC1 (28.99% and 13.24%, respectivelly) and PC2 (26.96% and 40.33%, respectivelly), reflecting only partial modulation of loop dynamics. In contrast, YM1 (PC1: 36.87% and PC2: 32.05%), which binds at the ZnF site rather than the BL2 pocket, produces a more isotropic variance profile, indicative of a distinct and less site-specific dynamical effect.

As highlighted above, our PCA analysis revealed that the apo system occupies a conformational region clearly separated from all ligand-bound systems in PC space, with the largest range and dispersion along PC1 (Fig. 5A). This broad sampling reflects a high degree of structural flexibility and suggests that, in the absence of ligand, PLpro explores a wide ensemble of conformations, consistent with the intrinsic mobility of regions such as the BL2 loop and the Zn-finger domain. Such behavior is compatible with an enzyme poised to access open and semi-open states that facilitate substrate recognition and binding. [41]

In contrast, all ligand-bound systems are displaced away from the apo ensemble and form more compact groups in the principal component space, indicating that ligand binding redirects the conformational landscape toward alternative, ligand-stabilized regions. Among these, XR3 (Fig. 5B) and A19 (Fig. 5C) exhibit the largest conformational volumes, showing that they do not simply rigidify the protein but instead allow it to explore multiple substates within a ligand-specific portion of the conformational space. This behavior suggests that potent BL2-site inhibitors can modulate the nature and directionality of collective motions – rather than uniformly reducing their amplitude – by stabilizing conformations that differ from those predominantly sampled by the apo form.

Weaker inhibitors such as CRY (Fig. 5D) and DIS (Fig. 5F) form a closely related cluster in PCA space and display intermediate conformational volumes, indicating that they shift the protein away from apo-like states but stabilize similar dynamical ensembles between themselves. This pattern is consistent with a partial modulation of protein flexibility. Notably, YM1 (Fig. 5E) shows the smallest conformational volume and a centroid displaced along PC2 relative to the other ligands, reflecting a distinct dynamical signature. This is in line with its binding at the ZnF region rather than the canonical BL2 allosteric pocket, leading to a more confined and different mode of motion that is dominated by ZnF–associated dynamics rather than BL2 loop rearrangements.

Recent quantum mechanics/molecular mechanics (QM/MM) simulations have elucidated the enzymatic mechanism of PLpro2, emphasizing the critical function of the catalytic triad and establishing the essential role of loop conformational dynamics during the acylation process [105]. These mechanistic findings underscore the significance of targeting regions with high conformational flexibility, such as the BL2 loop, which has been further validated through analyses of noncovalent inhibitors.

Altogether, the PCA and FEL analyses demonstrate that effective inhibition of PLpro2 is closely associated with diminished conformational plasticity and stabilization within a narrow, low-energy conformational basin. These findings point out the importance of loop immobilization, particularly within the BL2 region, as a structural hallmark of potent noncovalent inhibitors.

### MM/GBSA and SIE analysis

Accurately estimating binding free energy (ΔG_bind_) is essential for understanding how proteins and inhibitors interact, and it plays a key role in drug design [109]. There are several computational methods for this purpose. Rigorous approaches like thermodynamic integration (TI) [110] and free energy perturbation (FEP) [111] are highly accurate but require extensive computational resources and sampling, making them impractical for large-scale or high-throughput studies [112]. On the other hand, empirical scoring functions used in molecular docking are much faster but less accurate. [107]

A practical compromise is to use methods that incorporate features of both approaches, such as considering the configurational space and interactions in both bound and unbound states [113]. This hybrid strategy captures important energetic contributions while remaining computationally feasible [114, 115]. In our study, as detailed in the Materials and Methods section, we used the MM/GBSA and SIE end-point approaches, which have been successfully applied in SARS-CoV-2 research. [116–123]

**Fig. 6:**
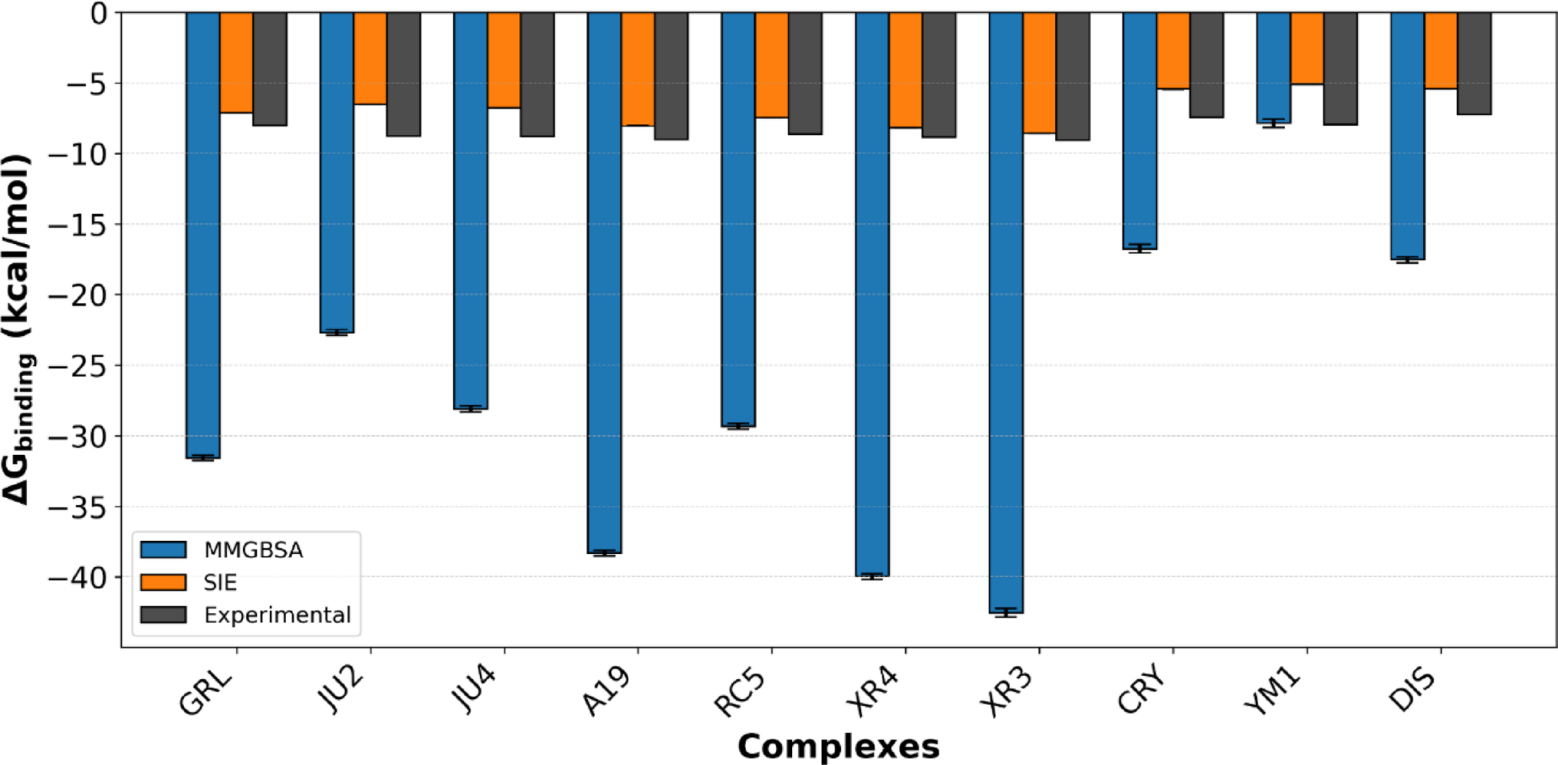
Comparative analysis of binding free energy (ΔG_bind_) for nine PLpro2 inhibitors. Bars represent values obtained from experimental measurements (black), SIE (orange) and MM/GBSA (blue)

Unlike previous studies, such as Ferreira et al. [41] and Sanachi et al. [124], which emphasize structural rearrangements, our approach integrates free energy decomposition and interaction fingerprints to map the energetic hotspots that stabilize the closed BL2 conformation. To elucidate the molecular basis underlying the inhibitory potency of selected noncovalent PLpro2 inhibitors, binding free energies were calculated for ten protein–ligand complexes using both the MM/GBSA and SIE methods. In light of the data presented in Table 4 and Fig. 6, the experimental binding free energies for the selected inhibitors—XR3, A19, YM1, CRY, and DIS—are − 9.04, − 9.02, − 7.96, − 7.45, and − 7.27 kcal/mol, respectively. This reflects a range of affinities that guided their selection for further analysis.

**Table 4:** Binding free energy (ΔG_bind_) and its components for the nine noncovalent inhibitors of PLpro2 evaluated in this study

System	XR3	A19	XR4	CRY	DIS	GRL	JU2	JU4	RC5	YM1
*Experimental*										
∆G_EXP_	–9.04	–9.02	–8.87	–7.45	–7.27	–8.03	–8.76	–8.81	–8.64	–7.96
*MM/GBSA*										
∆E_vdW_	–51.00 ± 0.02	–48.64 ± 0.05	–48.26 ± 0.02	–24.94 ± 0.03	–23.99 ± 0.03	–38.06 ± 0.02	–33.71 ± 0.02	–36.83 ± 0.02	–44.69 ± 0.02	–11.10 ± 0.02
∆E_elec_	–21.18 ± 0.03	–17.65 ± 0.05	–17.26 ± 0.02	–5.43 ± 0.03	–10.18 ± 0.02	–22.92 ± 0.04	–4.84 ± 0.02	–8.71 ± 0.02	–11.99 ± 0.02	–187.69 ± 0.19
∆G_GB_	36.36 ± 0.03	33.70 ± 0.05	31.21 ± 0.03	16.09 ± 0.03	19.74 ± 0.02	33.93 ± 0.03	19.61 ± 0.02	20.97 ± 0.02	30.75 ± 0.02	192.90 ± 0.19
∆G_nonpolar_	–5.84 ± 0.01	–5.70 ± 0.01	–5.47 ± 0.02	–2.50 ± 0.01	–3.12 ± 0.01	–4.52 ± 0.01	–4.11 ± 0.01	–4.17 ± 0.01	–5.44 ± 0.01	–2.60 ± 0.01
∆G_GBSA_	–42.54 ± 0.03	–38.30 ± 0.05	–39.98 + -0.02	–16.78 ± 0.03	–17.55 ± 0.02	–31.57 ± 0.02	–23.05 ± 0.02	–28.74 ± 0.02	–31.36 ± 0.02	–8.48 ± 0.03
*SIE*										
E_vdW_	–51.8 ± 4.14	–48.64 ± 0.07	–48.25 ± 3.50	–24.94 ± 0.05	–24.00 ± 0.04	–38.05 ± 2.70	–34.35 ± 2.71	–36.54 ± 0.04	–43.63 ± 0.04	–11.12 ± 0.03
E_coul_	–9.41 ± 2.70	–7.84 ± 0.03	–7.77 ± 2.32	–2.42 ± 0.02	–4.53 ± 0.02	–10.19 ± 3.10	–2.33 ± 1.66	–4.32 ± 0.02	–5.25 ± 0.02	–83.42 ± 0.12
E_RF_	15.57 ± 2.65	15.01 ± 0.03	13.31 ± 2.89	7.09 ± 0.02	7.09 ± 0.02	14.32 ± 3.02	7.74 ± 1.91	9.83 ± 0.02	12.26 ± 0.02	76.64 ± 0.11
E_nonpolar_	–8.47 ± 0.68	–7.86 ± 0.01	–8.01 ± 0.00	–4.07 ± 0.01	–4.07 ± 0.01	–6.63 ± 0.44	–5.90 ± 0.40	–6.18 ± 0.01	–7.32 ± 0.01	–3.31 ± 0.01
∆G_SIE_	–8.57 ± 0.54	–8.06 ± 1.04	–8.20 ± 0.44	–5.44 ± 00.61	–5.44 ± 0.54	–7.14 ± 0.32	–6.54 ± 0.31	–6.79 ± 0.44	–7.49 ± 0.40	–5.11 ± 0.53

Experimental values (ΔG_EXP_) alongside those predicted by the SIE (ΔG_SIE_) and MM/GBSA (ΔG_GBSA_) methods. A single-trajectory scheme [96] was used to compute the average and SEM for each system. All values are reported in kcal/mol

Initially, XR3 and A19, the two most potent inhibitors based on experimental ΔGbind (–9.04 and –9.02 kcal/mol, respectively), exhibit the most favorable binding profiles in MM/GBSA and SIE calculations. XR3 is driven by the strongest van der Waals (–51.88 kcal/mol) contributions. A19 similarly shows a highly favorable van der Waals (–48.65 kcal/mol). SIE values also corroborate these trends, with XR3 (–8.51 kcal/mol) and A19 (–7.44 kcal/mol) showing the lowest predicted ΔG_bind_ among the group.

YM1 is an outlier among the inhibitors analyzed. Its experimental binding free energy is moderately favorable at –7.96 kcal/mol; however, the MM/GBSA and SIE methods significantly underestimate its affinity, predicting values of –8.48 and –5.07 kcal/mol, respectively. This discrepancy is primarily due to an exceptionally high solvation penalty (ΔG_solv_ =  + 190.31 kcal/mol), which counteracts the otherwise favorable gas-phase interaction energy (ΔG_gas_ = –198.79 kcal/mol).

The significant electrostatic contribution (ΔE_elec_ = –187.69 kcal/mol) indicates strong polar interactions between YM1 and the PLpro2 binding site. However, these interactions are susceptible to desolvation effects, which may be overestimated in implicit solvent models, such as GB. Additionally, a dual binding mode observed in experimental structural data [51] could further complicate the energy landscape, introducing conformational heterogeneity that is not adequately captured by single-structure endpoint methods, such as MM/GBSA and SIE.

In contrast, CRY and DIS, the least potent inhibitors based on experimental binding free energies (–7.45 and –7.27 kcal/mol, respectively), exhibit consistently weak energetic profiles across computational analyses. Their gas-phase interaction energies (ΔG_gas_ = –30.37 and –34.16 kcal/mol, respectively) are the least favorable among the selected inhibitors, primarily due to limited van der Waals stabilization (–24.94 and –23.99 kcal/mol, respectively). Interestingly, DIS shows a relatively strong electrostatic contribution (ΔE_ele_ = –18.18 kcal/mol), comparable to those of more potent inhibitors such as A19 (–17.64 kcal/mol) and XR3 (–21.18 kcal/mol). However, in the case of DIS, this electrostatic component is not synergistically reinforced by favorable van der Waals interactions or effective shape complementarity within the binding pocket. As a result, the net gas-phase stabilization remains insufficient to drive high-affinity binding. The moderate solvation penalties for CRY and DIS (ΔG_solv_ =  + 13.59 and + 16.61 kcal/mol, respectively) do not provide a compensatory effect to improve binding energetics. This leads to unfavorable overall MM/GBSA binding estimates. The ΔG_SIE_ for CRY (–5.40 kcal/mol) and DIS (–5.39 kcal/mol) indicate low affinity, further confirming their weak inhibitory profiles. These findings underscore that while strong electrostatic interactions are necessary, they are insufficient in isolation to ensure high binding affinity, especially in the absence of complementary van der Waals contacts and efficient desolvation.

Both approaches accurately identified potent inhibitors, such as XR3 and A19, as high-affinity binders. However, the SIE method demonstrated a stronger qualitative correlation with experimental free energy values compared to MM/GBSA (Fig. 6). This improved correlation may be attributed to MM/GBSA's inherent approximations and sensitivity to solvation models, which can result in overestimation of absolute binding free energies [125, 126]. Despite these findings, the theoretical ranking of inhibitors frequently diverges from the experimental potency order. Certain compounds predicted as weak binders by MM/GBSA or SIE exhibit strong experimental inhibition, whereas others display the opposite trend [127–129]. Such discrepancies may result from limitations inherent in the scoring models, incomplete accounting of entropic contributions in endpoint methods, or unmodeled factors such as binding kinetics and induced fit effects [130, 131]. Consequently, while computational free energy methods provide valuable trend estimations, their results must be interpreted cautiously and supported by additional structural and dynamic analyses, including PCA, FEL mapping and IFP, as implemented in this study [96]. 

**Fig. 7:**
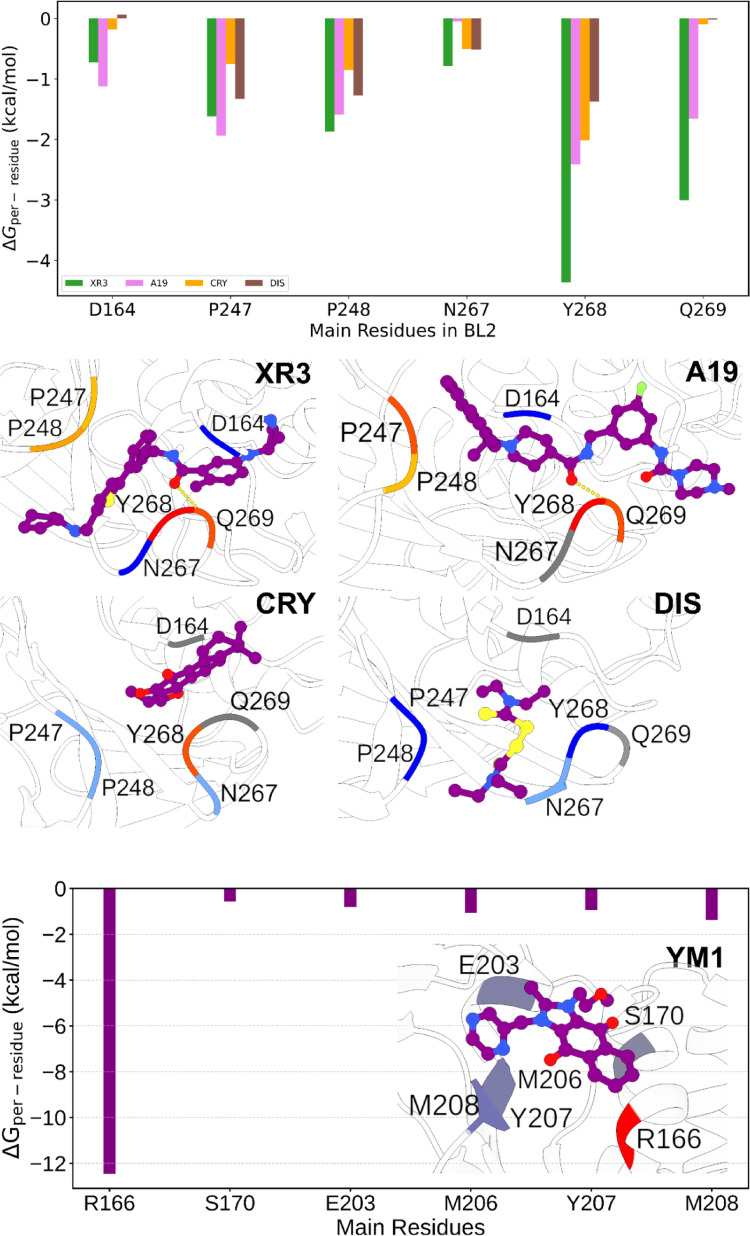
Per-residue energy decomposition analysis of PLpro2 complexes with five noncovalent inhibitors. The upper panels correspond to XR3, A19, CRY, and DIS, while the lower panel shows the YM1 complex

While a rigorous decomposition of binding free energy into individual residue contributions is not theoretically precise within classical MD frameworks [132, 133], structural and interaction analyses can offer valuable qualitative insights [122, 134–138]. We compared their binding modes to gain a deeper understanding of how selected noncovalent inhibitors interact with PLpro2. Key protein–ligand contacts were identified and mapped across the five representative complexes, providing complementary information to the energy-based evaluations (Fig. 7).

Our energy decomposition analysis identifies several hotspot residues that consistently enhance binding affinity across the inhibitor series (Fig. 7). Notably, Tyr268 and Gln269, situated within the BL2 loop, are principal contributors, especially in complexes with high-affinity ligands XR3 and A19. Tyr268 primarily participates in stable π–π stacking interactions, contributing –4.37 kcal/mol (XR3) and –2.41 kcal/mol (A19), while Gln269 forms persistent hydrogen bonds, with contributions of –3.00 kcal/mol (XR3) and –1.66 kcal/mol (A19). Both residues exhibit substantially more favorable energetic contributions (ΔE < –2 kcal/mol) in the context of potent inhibitors relative to weaker binders such as CRY (Tyr268: –2.02 kcal/mol; Gln269: –0.10 kcal/mol) and DIS (Tyr268: –1.37 kcal/mol; Gln269: –0.01 kcal/mol), and especially YM1, which shows negligible stabilization (Tyr268: 0.01 kcal/mol; Gln269: 0.01 kcal/mol). Our findings extend previous results [41] by providing residue-level energetic insights into the stabilization of the BL2-loop. Specifically, we observed that π-stacking with Tyr268 and hydrogen bonding with Gln269 align with the inhibitor-induced closed state reported in Markov state model state S4 (π = 0.529), as reported by Ferreira et al. [41].

Gly266, located in the BL2 loop, also contributes favorably to binding energetics, corroborating its role in stabilizing the loop conformation upon inhibitor engagement. In the cases of A19 and XR3, the aggregate contribution of BL2 loop residues—Gly266, Asn267, Tyr268, and Gln269—is pronounced, further underscoring the critical role of loop closure as a determinant of binding affinity.

On the other hand, CRY and DIS exhibit diminished or negligible stabilization from these key residues, indicating either increased loop flexibility or suboptimal interaction geometries within their respective binding conformations. In particular, YM1 shows minimal energetic contributions from Tyr268 and Gln269, consistent with its atypical interaction patterns observed in PCA, FEL projections, and ProLIF assessments. It should be noted that, unlike other inhibitors, YM1 exhibits a unique binding profile, interacting with multiple regions of PLpro beyond the BL2 pocket [51]. Consequently, YM1 does not induce sustained BL2-loop stabilization and is considered a mechanistic outlier within the current inhibitor set. For inhibitors located in the BL2 region, Asp164 residue, positioned proximal to the catalytic triad, displays moderate favorable energetic contributions across all inhibitor complexes, likely attributable to electrostatic interactions with polar functional groups on the inhibitors. Additionally, Pro247 and Pro248, which form part of the hydrophobic pocket within the binding groove, contribute more substantially in the XR3 and A19 complexes (Pro247: –1.622 and –1.934 kcal/mol; Pro248: –1.872 and –1.586 kcal/mol, respectively), suggesting enhanced insertion of aromatic or nonpolar moieties into this region.

These findings underscore that high-affinity inhibitors exploit a synergistic network of noncovalent interactions—particularly those mediated by Tyr268, Gln269, and adjacent residues—to stabilize the closed conformation of PLpro2. The energetic contributions outlined here are consistent with structural observations from ProLIF and FEL analyses, providing a quantitative framework for identifying the residues most critical to binding optimization.

**Fig. 8:**
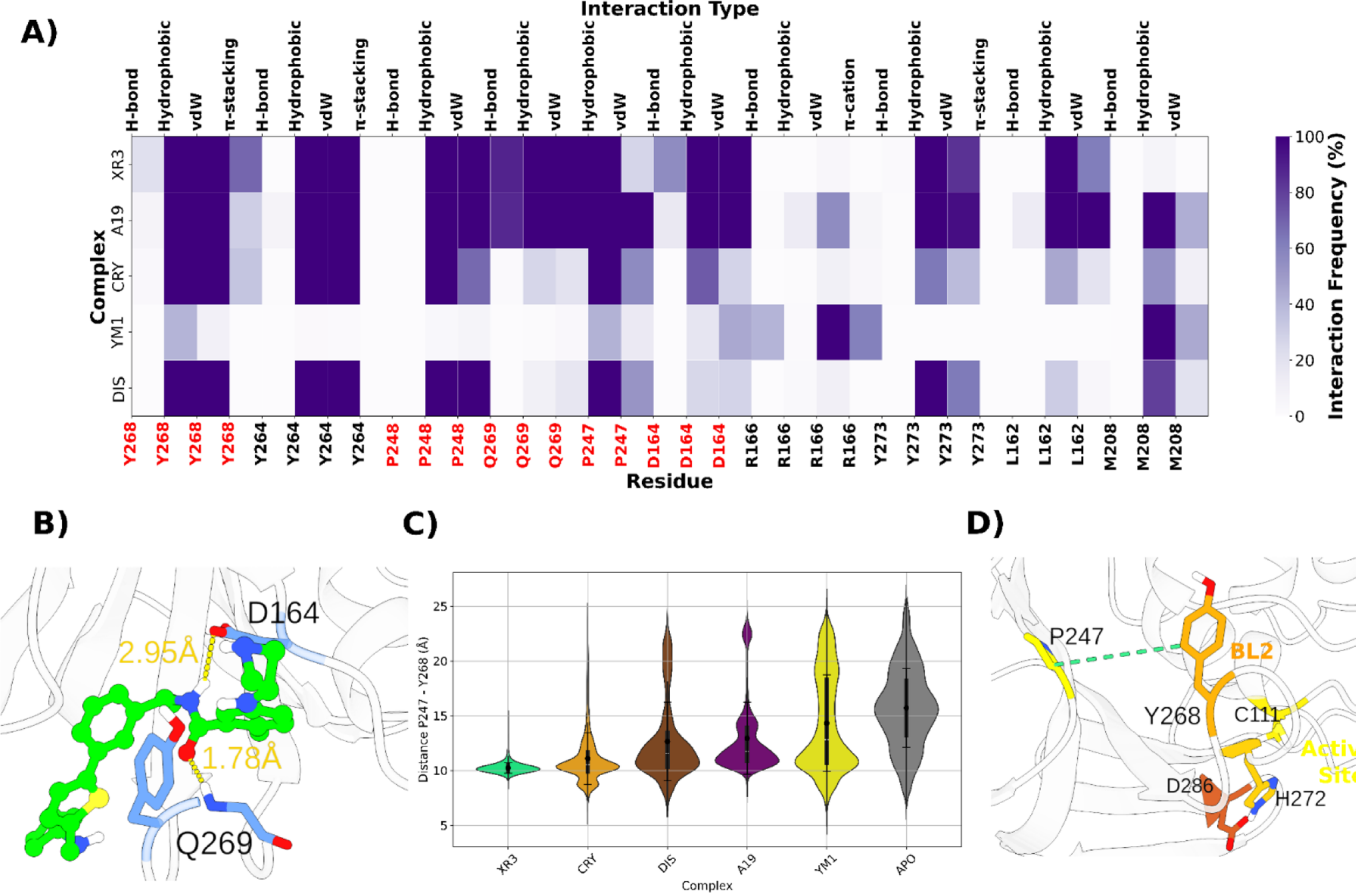
**A** Interaction fingerprint heatmap showing the average residue–ligand contacts for five PLpro2 inhibitor complexes (A19, XR3, CRY, DIS, YM1). Interaction frequencies were calculated over the entire simulation time and averaged across three independent MD replicas. **B** Representative snapshot of the PLpro2–XR3 complex highlighting key hydrogen-bond interactions with Asp164 and Gln269. **C** Distribution plot of the inter-residue distances between Pro247 and Tyr268. **D** Representative 3D conformation illustrating the interaction between Pro247 and Tyr268

The ProLIF-based interaction analysis elucidated distinct residue engagement profiles among the five PLpro inhibitors evaluated (Fig. 8A). Notably, A19 and XR3, which exhibited the highest potency based on both experimental and computational binding affinities, demonstrated robust and persistent interactions with critical residues within the BL2 region (Fig. 8B). Specifically, these compounds engaged Tyr268 through π–π stacking and Gln269 via stable hydrogen bonding. Such interactions are recognized as key determinants in stabilizing the closed conformation of the BL2 loop, thereby impeding substrate access to the catalytic triad (Fig. 8C and D).

Whereas, DIS and CRY displayed weaker and less frequent interactions with these critical residues. Their binding profiles were predominantly characterized by transient hydrophobic contacts with peripheral residues, such as Pro247 and Tyr264, yet lacked sustained engagement with the BL2 loop anchoring residues. This interaction deficiency is consistent with their elevated ΔG_bind_ values and diminished experimental potency.

Intriguingly, YM1 exhibited a unique interaction pattern, characterized by moderate occupancy at residues located outside the canonical BL2 binding cleft. This observation suggests the possibility of an alternative binding pose or allosteric modulation mechanism. Such atypical binding behavior may also account for its suboptimal performance in free energy calculations, as conventional scoring functions may inadequately capture non-canonical binding contributions.

These findings substantiate the hypothesis that π-stacking interactions with Tyr268 and hydrogen bonding with Gln269 constitute hallmark features of high-affinity PLpro inhibitors. The capacity of A19 and XR3 to simultaneously establish these contacts underpins their superior binding profiles. In contrast, the absence or instability of such interactions in DIS, CRY, and YM1 is correlated with reduced inhibitory activity. These observations align with structural models advanced by Ferreira et al. [41] wherein BL2-loop closure was identified as a critical determinant of inhibitor efficacy.

Moreover, our results of this study align with previous computational and experimental investigations that evaluated both covalent and noncovalent PLpro2 inhibitors, notably those identifying Tyr268 and Gln269 as critical residues for ligand binding [139]. In contrast to earlier reports, the present work employs principal component analysis, free energy landscape modeling, and protein–ligand interaction fingerprinting to deliver a more holistic perspective on the influence of ligand-induced conformational restriction on inhibitory efficacy. This integrated approach advances the understanding of molecular determinants underlying potent PLpro2 inhibition.

## Conclusions

This study presents a comprehensive investigation into the structural and energetic mechanisms underlying noncovalent inhibition of PLpro2. Utilizing molecular docking, multi-replica MD simulations, MM/GBSA and SIE binding free energy calculations, as well as advanced dynamic analyses including PCA and FEL mapping, we elucidated distinct conformational and interaction signatures correlating with inhibitor potency. High-affinity inhibitors, exemplified by XR3 and A19, were observed to markedly restrict the conformational flexibility of PLpro2—particularly within the BL2 loop—and to establish robust, persistent interactions with key residues Tyr268 and Gln269, which serve as critical anchors for loop closure. These results were further substantiated by energy decomposition and ProLIF interaction fingerprinting, which identified Tyr268 π-stacking and Gln269 hydrogen-bonding as defining features of potent ligand engagement.

In opposition, weakly binding inhibitors such as DIS, CRY, and YM1 were unable to stabilize closed-loop conformations, exhibited diffuse free energy landscapes, and failed to form persistent interactions within the primary binding cleft. Our findings underscore that effective noncovalent inhibition is contingent not only upon active site occupancy, but also upon the induction of structural rigidity through targeted residue engagement.

Therefore, our study provides an useful framework for the rational design of next-generation PLpro2 inhibitors. The critical findings include: (i) the identification of key interaction hotspots, such as Tyr268 and Gln269 in the BL2 loop, which are involved in high-frequency π–π stacking and hydrogen bonding with potent ligands; (ii) mechanistic analyses using PCA, FEL and RMSF demonstrate that effective inhibitors decrease structural flexibility and stabilize the BL2 loop in a closed, inactive conformation, thereby blocking substrate access; (iii) energetic profiling through MM/GBSA and SIE calculations with decomposition analysis mapped residues with the greatest contribution to binding free energy, aiding in rational scaffold optimization; (iv) additionally, integration of IFP using ProLIF-based maps revealed ligand-specific contact patterns, which inform functional group placement to enhance affinity and selectivity.

## Supplementary Information

Below is the link to the electronic supplementary material.

Supplementary file1 (PDF 846 kb)

## Data Availability

Topologies, coordinates, data and scripts are publicly available on GitHub: [https://github.com/fvtargaryen/PLpro2-noncovalent-systems] (https://github.com/fvtargaryen/PLpro2-noncovalent-systems).
