# Supplementary material for: Mechanistic insights into the noncovalent inhibition of SARS-CoV-2 PLpro: a multiscale computational study
Source: J Comput Aided Mol Des. 2026 Feb 5;40(1):56. doi: 10.1007/s10822-026-00763-z (PMC12872634; doi:10.1007/s10822-026-00763-z)

Mechanistic Insights into the Non-Covalent Inhibition of SARS-CoV-2 PLpro: A Multiscale Computational Study

Flávio Vinícius da Silva Ribeiro, Renan Patrick da Penha Valente, Hendrik G. Kruger, Jéssica de Oliveira Araújo and José Rogério A. Silva

Supporting Information

Table S1. RMSD values for the crystallographic complexes indicate that Molegro Virtual Docker is the best software for simulations of naphthalene inhibitors in PLpro2

System	VINA	ADT	MVD	GOLD	DOCK	HYBRID
GRL	0.14	0.49	0.13	0.33	0.39	0.68
JU2	0.19	1.83	0.20	0.46	0.38	0.41
XR4	0.77	0.92	0.30	0.66	0.32	0.15
YM1	0.94	0.67	0.21	0.31	6.72	0.75

Table S2. RMSD and RMSF values of all PLpro2 systems.

Plpro2 system	RMSD (Å)	RMSF (Å)
GRL	1.45±0.3	1.27±0.74
JU2	1.37±0.27	1.17±0.74
JU4	1.37±0.27	1.19±0.72
A19	1.43±0.28	1.24±0.75
RC5	1.45±0.42	1.30±0.77
XR3	1.83±0.31	1.18±0.71
XR4	1.57±0.38	1.38±0.83
CRY	1.39±0.28	1.21±0.74
YM1	1.38±0.26	1.18±0.74
DIS	1.48±0.29	1.28±0.80
APO	1.88±0.33	1.29±0.79

Figure S1. Molecular docking interactions for non-crystallographic complexes. A) XR3, B) JU4, C) RC5, D) A19, E) CRY and F) DIS.

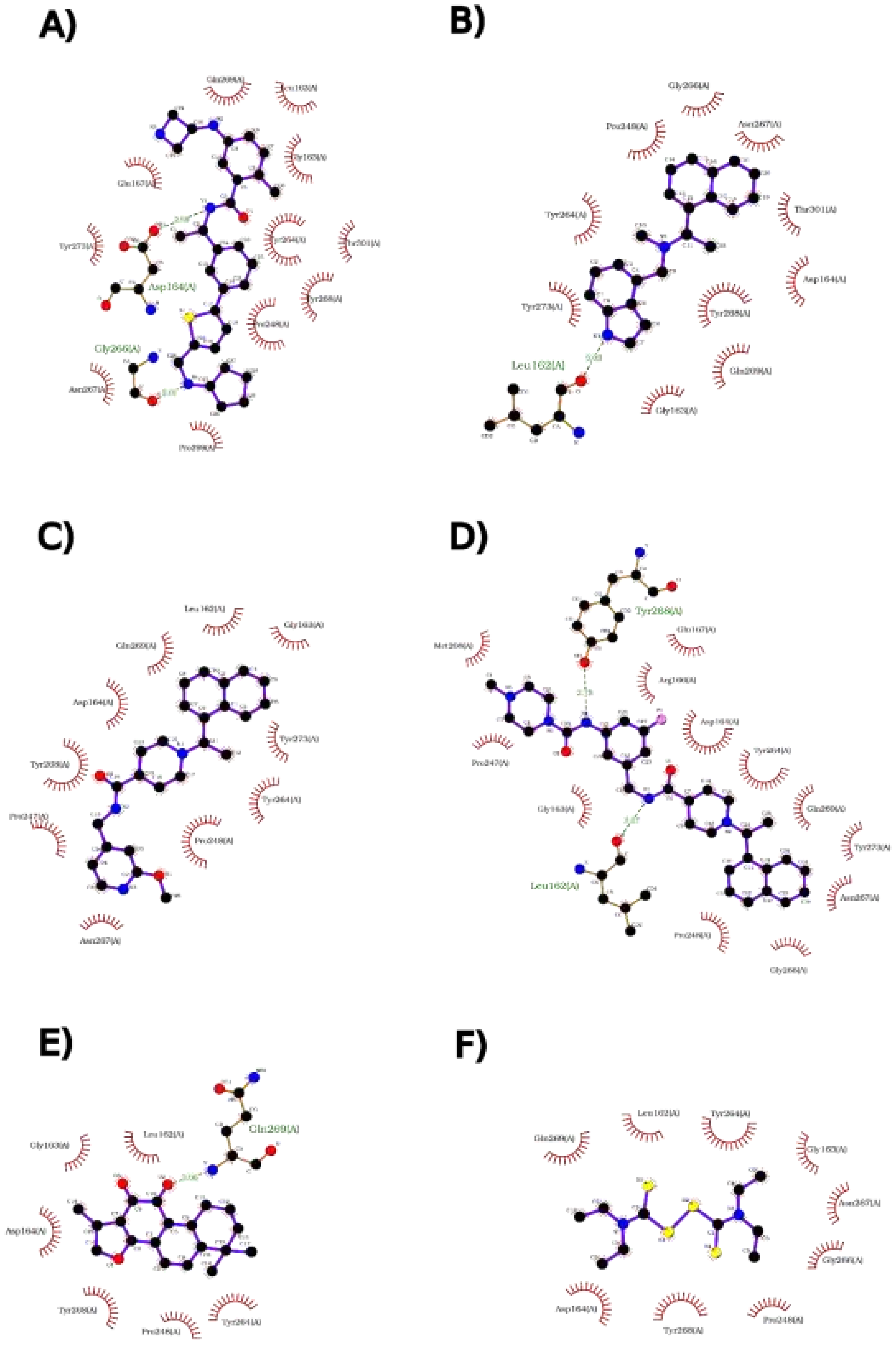

Figure S2. RMSD plots for each PLpro2 system (3 replicas x 500 ns of MD).

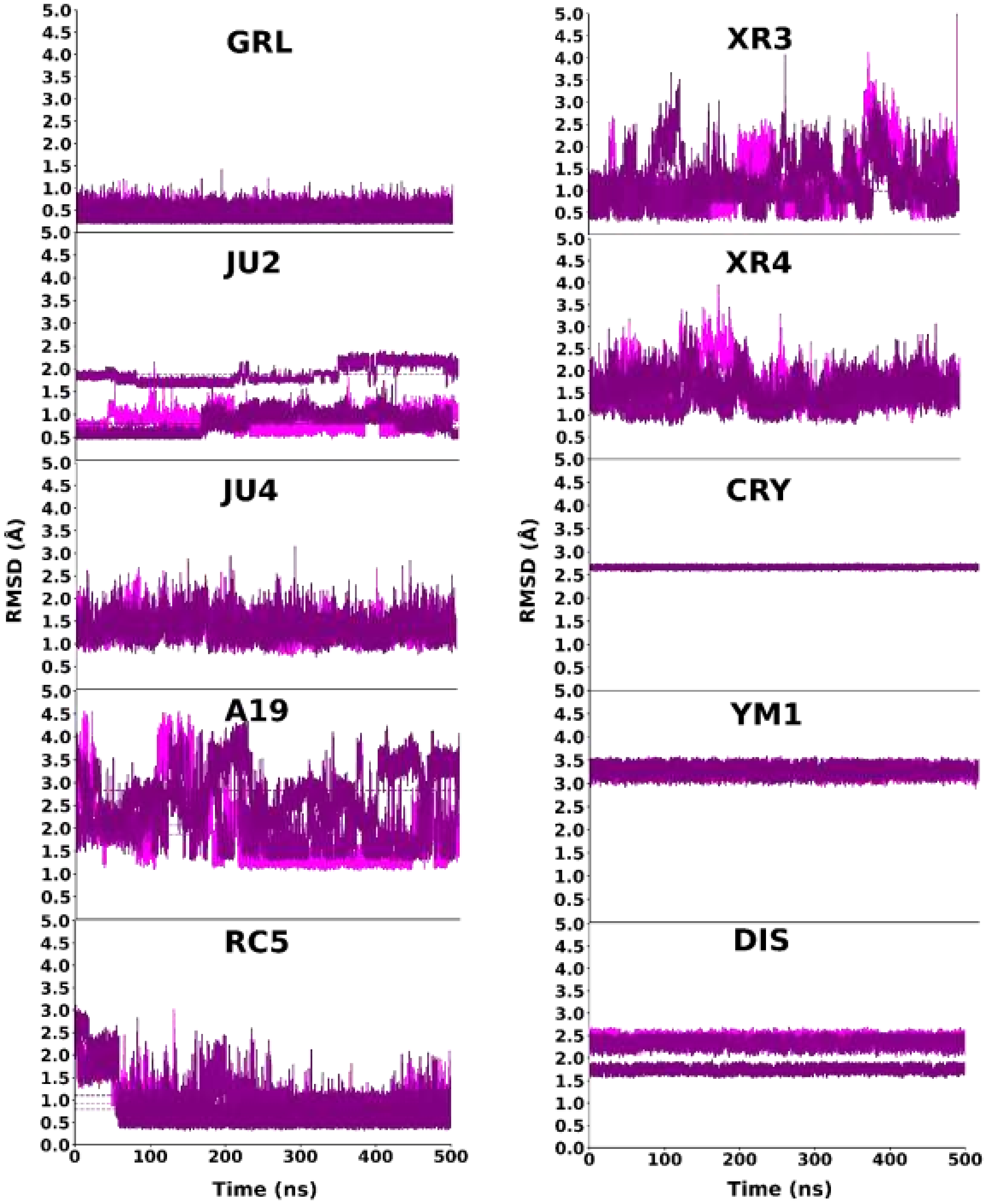

Figure S3. RMSF plots for each PLpro2 system (3 replicas x 500 ns of MD).

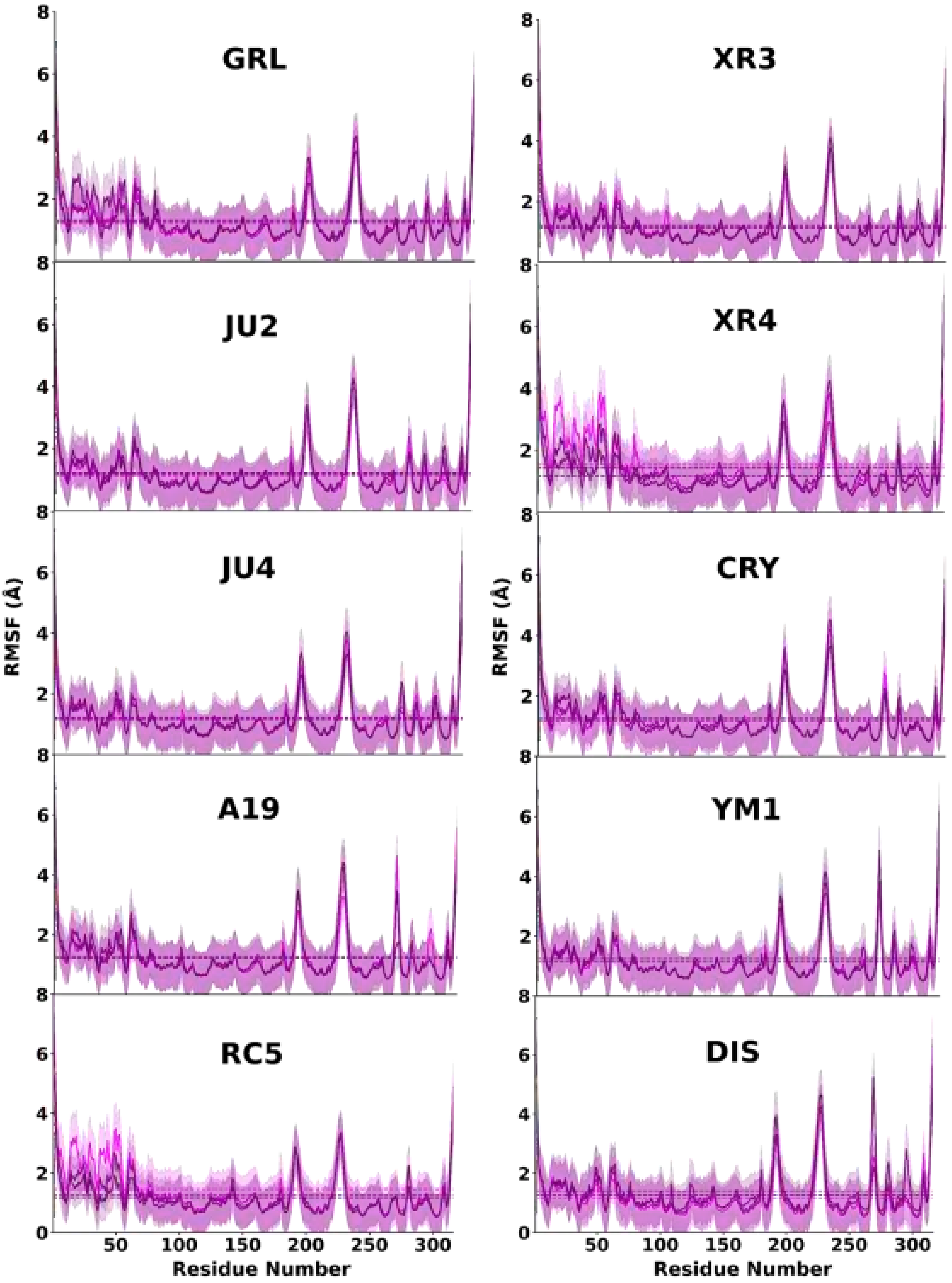

PCA was conducted to characterize the dominant collective motions of PLpro in the presence of various inhibitors. The first principal component (PC1) describes large-amplitude motions associated with the opening and closing of the BL2 loop, which modulate access to the substrate-binding pocket. In contrast, the second principal component (PC2) captures localized fluctuations involving loop rearrangements and relative movements between the thumb and palm domains. Collectively, these components explain most of the conformational variance observed in the simulations and offer a mechanistic framework for interpreting inhibitor-dependent stabilization or destabilization of the BL2 loop.

Figure S4. PCA and FEL plots for each PLpro2 system (3 replicas x 500 ns of MD). A) GRL, B) JU2, C) JU4, D) RC5 and E) XR4.

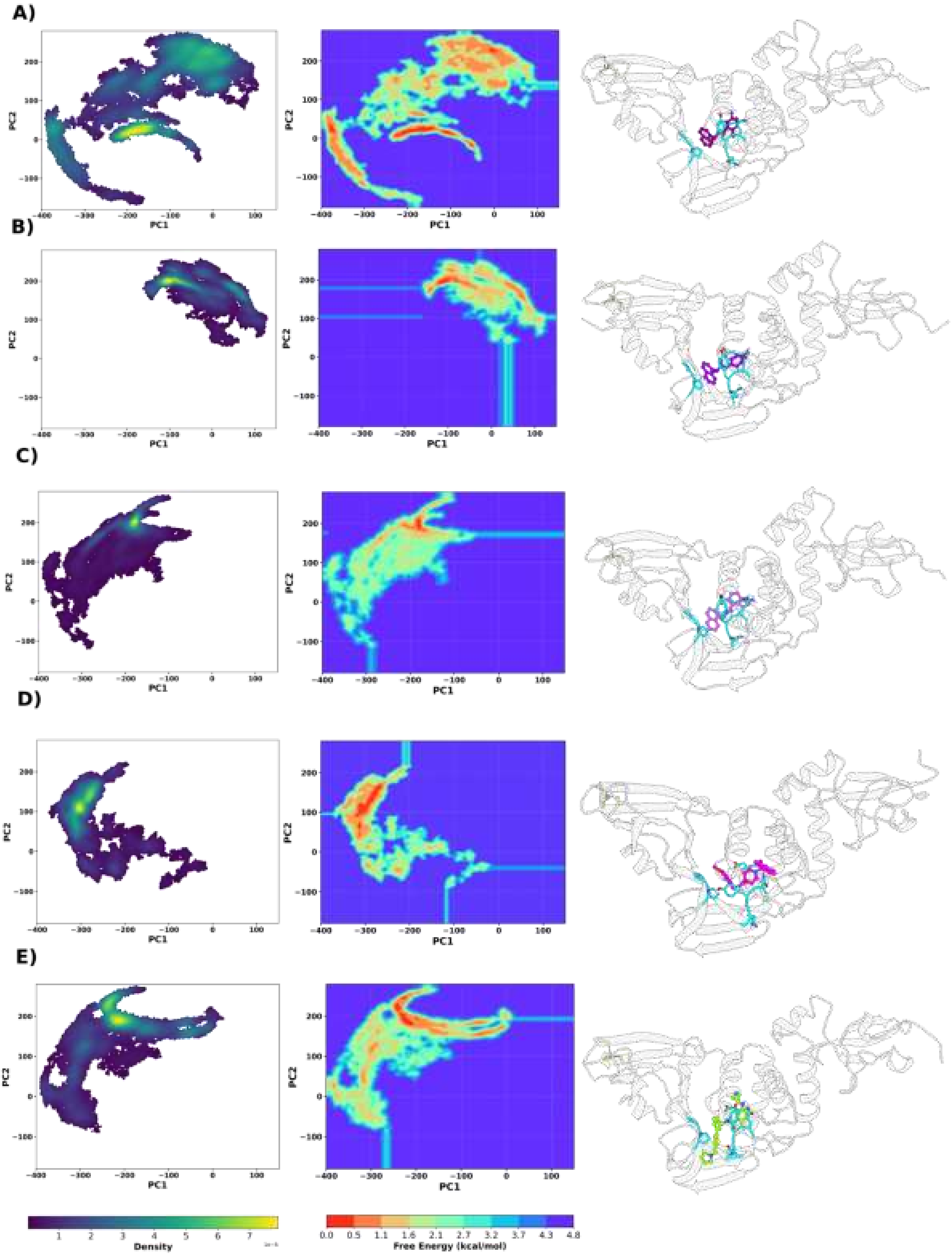
A)
B)
C)
D)
E)
PC1
PC2
Density
Free Energy (kcal/mol)
0.0 0.5 1.1 1.6 2.1 2.7 3.2 3.7 4.3 4.8

Figure S5. Residual decomposition results for the remaining five PLpro2 inhibitor complexes (GRL, JU2, JU4, XR4, and RC5).

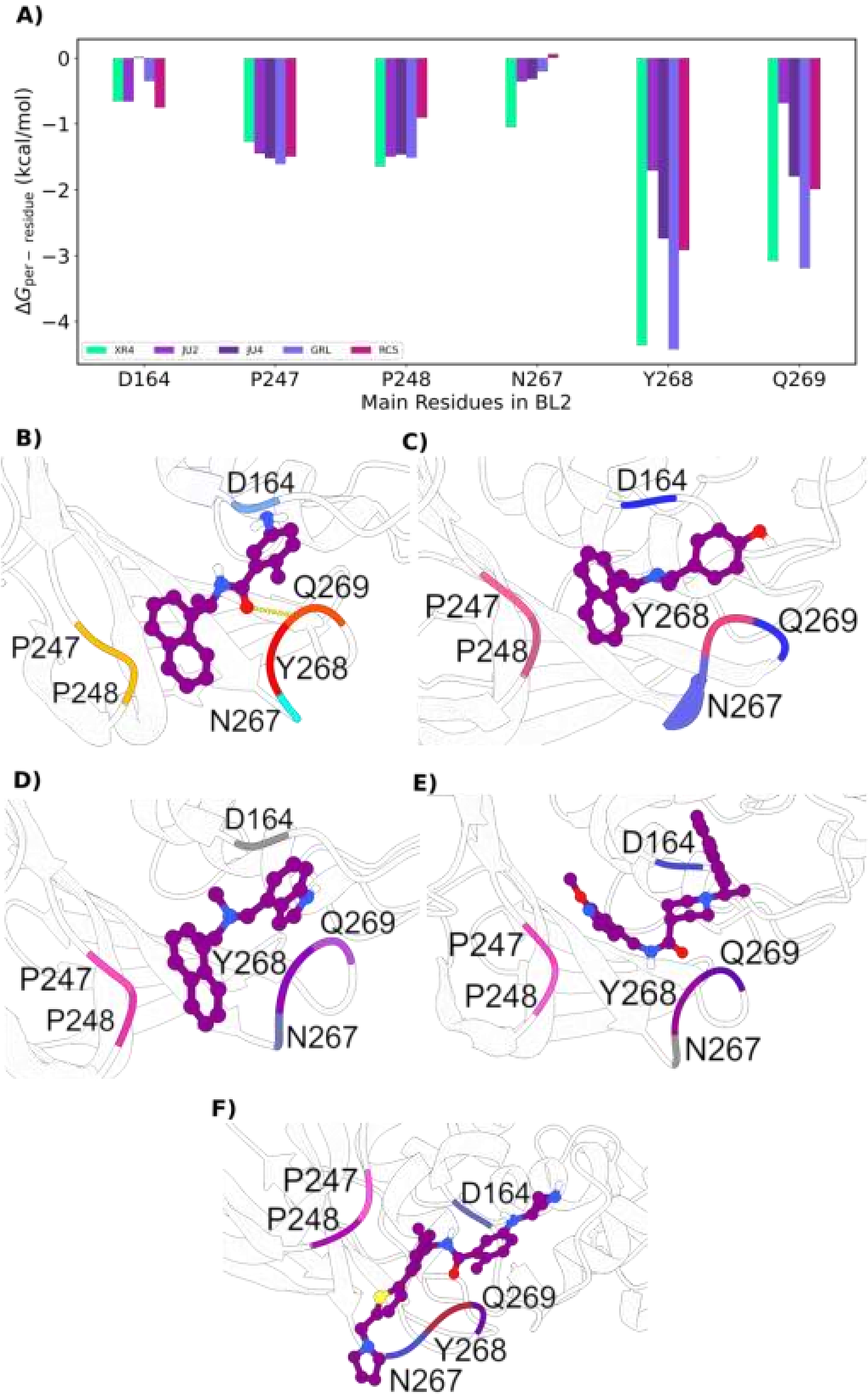

Figure S6. Heatmap of protein–ligand interaction fingerprints for the remaining five PLpro2 inhibitor complexes (GRL, JU2, JU4, XR4, and RC5), which exhibited intermediate binding energies. Higher interaction frequencies indicate more persistent and stable contacts, providing insight into the binding profiles of moderately potent inhibitors.

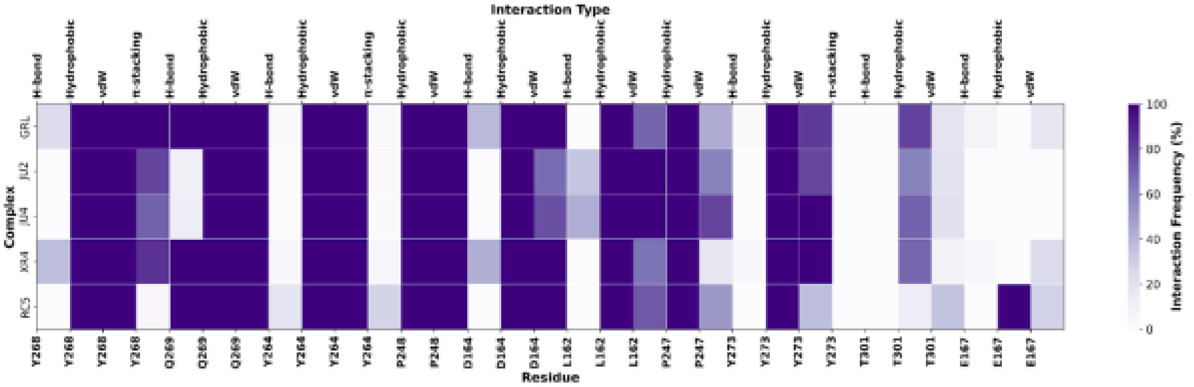

Figure S7. Secondary structure of PLpro2 based on PDB entry 7LBS, highlighting the catalytic triad residues (Cys111, His272, and Asp286) with red boxes.

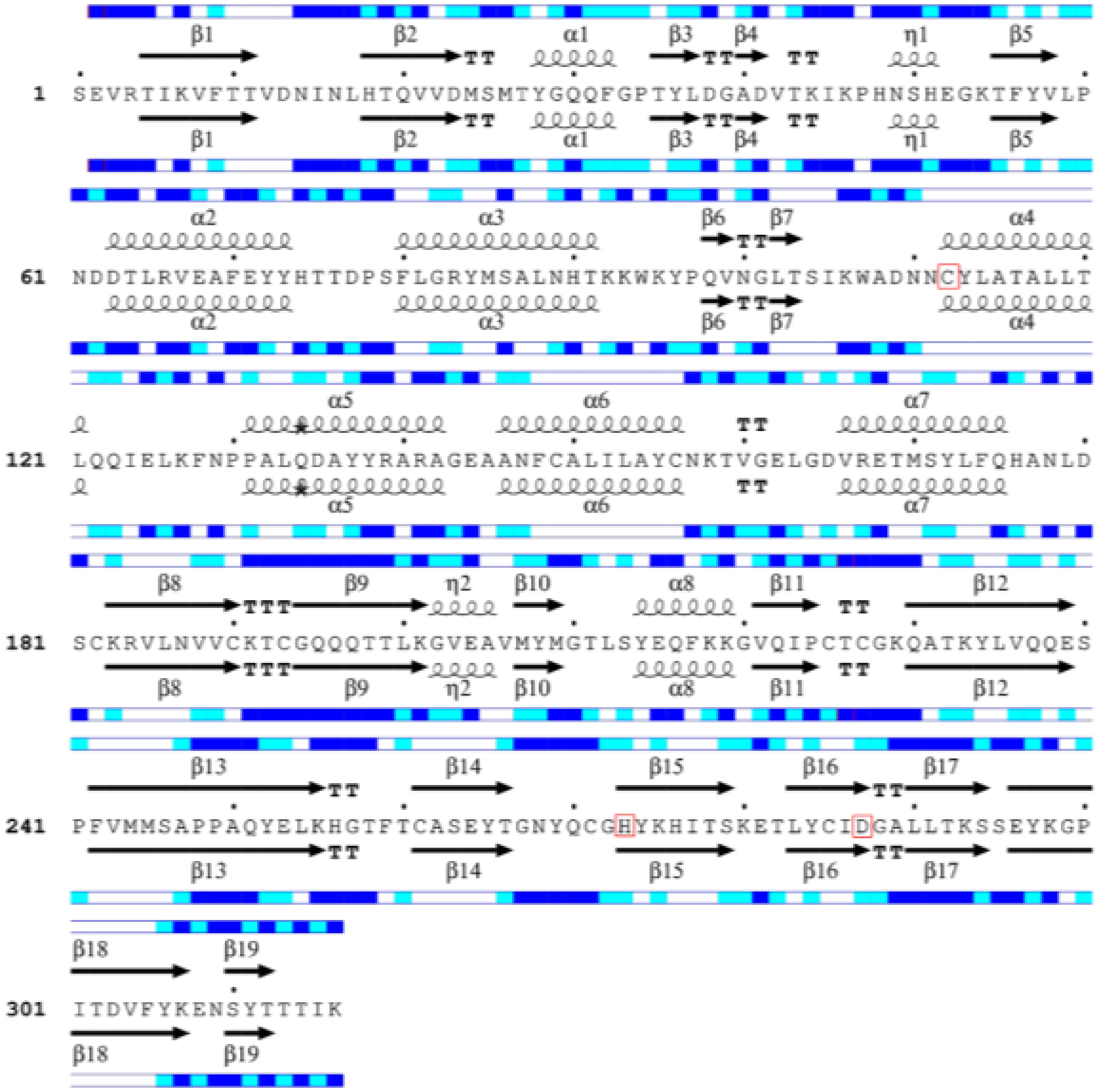

Supplement: Supplementary file 1 — Supplementary file1 (PDF 846 kb) [file 10822_2026_763_MOESM1_ESM.pdf]
